# Zoliflodacin versus ceftriaxone plus azithromycin for treatment of uncomplicated urogenital gonorrhoea: an international, randomised, controlled, open-label, phase 3, non-inferiority clinical trial

**DOI:** 10.1016/S0140-6736(25)01953-1

**Published:** 2026-01-10

**Authors:** Alison Luckey, Manica Balasegaram, Lindley A Barbee, Teresa A Batteiger, Helen Broadhurst, Stephanie E Cohen, Sinead Delany-Moretlwe, Henry J C de Vries, Jodie A Dionne, Katherine Gill, Chris Kenyon, Rossaphorn Kittiyaowamarn, Drew Lewis, John P Mueller, Vimla Naicker, Seamus O'Brien, John P O'Donnell, Nittaya Phanuphak, Elizabeth Spooner, Subasree Srinivasan, Stephanie N Taylor, Magnus Unemo, Zinhle Zwane, Edward W Hook, Zoliflodacin Phase 3 Study Group, Keisha De Gouveia, Thembisa Makowa Mkhize, Samantha Siva, Lindy Gumede, Ranmini Kularatne, Venessa Maseko, Shabashini Reddy, Patience Kwedza, Ravesh Singh, Lisha Sookan, Danielle Travill, Kittipoom Chinhiran, Sarinthorn Mongkolrat, Chatnapa Duangdee, Jantawan Satayarak, Siriporn Nonenoy, Supanat Thitipatarakorn, Joseph V Woodring, Wannee Chonwattana, Supawadee Na-pompet, Waropart Pongchaisit, Suwan Sriviriyakul, Tanyaporn Wansom, Aaron Ermel, Lora Fortenberry, Catherine L Cammarata, Rebecca Lillis, Alison Cohee, Ejovwoke Dosunmu, Godfred Masinde, Paula Dixon, Julia C Dombrowski, Olusegun O Soge, Elske Hoornenborg, Alje van Dam, Vicky Cuylaerts, Irith Debaetselier, Angèle Gayet-Ageron, Sarah M. McLeod, Alita Miller, Sarah Cohen, Hilary Johnstone, Lebogang Tshehla, Emilie Alirol, Carmen Au, Cherine Bajjali, Esther Bettiol, Pierre Daram, Amalia Droal, Varalakshmi Elango, Christophe Escot, Markus Heep, Karin Hergarden, Daniel Iniguez, Gabrielle Kornmann, Jean-François Louvion, Manon Manuelli, Jessica Renaux, Mary-Ann Richardson

**Affiliations:** a Global Antibiotic Research & Development Partnership, Geneva, Switzerland; b University of Washington, Seattle, WA, USA; c Indiana University School of Medicine, Indianapolis, IN, USA; d Plus-Project, Knutsford, UK; e San Francisco Department of Public Health, San Francisco, CA, USA; f Wits RHI, University of the Witwatersrand, Johannesburg, South Africa; g Department of Dermatology, Amsterdam UMC, University of Amsterdam, Amsterdam, Netherlands; h Amsterdam Institute for Infection and Immunity, Infectious Diseases, Amsterdam, Netherlands; i Center for Sexual Health, Department of Infectious Diseases, Public Health Service Amsterdam, Netherlands; j Amsterdam Institute for Global Health and Development, Amsterdam, Netherlands; k University of Alabama at Birmingham, Birmingham, AL, USA; l Desmond Tutu HIV Centre, University of Cape Town, Cape Town, South Africa; m Institute of Tropical Medicine, Antwerp, Belgium; n Bangrak STIs Center, Division of AIDS and STIs, Department of Disease Control, Ministry of Public Health, Bangkok, Thailand; o Innoviva Specialty Therapeutics, Waltham, MA, USA; p HIV and Other Infectious Diseases Research Unit, South African Medical Research Council, Durban, South Africa; q Institute of HIV Research and Innovation, Bangkok, Thailand; r Louisiana State University Health Sciences Center, New Orleans, LA, USA; s WHO Collaborating Centre for Gonorrhoea and Other STIs, Örebro University, Örebro, Sweden; t Institute for Global Health, University College London, London, UK; u Setshaba Research Centre, Soshanguve, South Africa

## Abstract

**Background:**

Development of new treatments for gonorrhoea is a global public health priority. We aimed to evaluate the efficacy and safety of zoliflodacin versus ceftriaxone plus azithromycin in patients with uncomplicated urogenital gonorrhoea.

**Methods:**

In this phase 3, multinational, randomised, controlled, open-label, non-inferiority clinical trial, participants aged 12 years and older with clinical suspicion of uncomplicated urogenital gonorrhoea were eligible for inclusion. The trial was done in 17 outpatient clinics in Belgium, the Netherlands, South Africa, Thailand, and the USA. Participating countries with high disease prevalence were identified for participation in the study. Sites selected for participation were led by principal investigators with research experience, who were knowledgeable in HIV or sexually transmitted infections and treatment. Feasibility questionnaires and prestudy visits assessed sexually transmitted infection case management guidelines, clinical services, and resources (ie, facility, staff, proposed composition of the study team, standard sexually transmitted infection services offered at the site, assessment of laboratory capacity, research experience and ethical review of clinical trials). Eligible participants were randomly assigned (2:1) to receive a single dose of zoliflodacin 3 g (oral) or ceftriaxone 500 mg (intramuscular) plus azithromycin 1 g (oral). Treatment assignment was known to the participants and their treating clinicians; however, microbiology laboratory staff were masked and the sponsor's central study team were masked until after database lock. The primary endpoint was the proportion of patients with microbiological cure (eradication of *Neisseria gonorrhoeae*, determined by urethral or endocervical culture) at test of cure (TOC; day 6 ± 2) in the microbiological intention-to-treat population. The primary efficacy analysis declared non-inferiority if the upper bound of the two-sided 95% CI for the treatment difference (comparator minus zoliflodacin) fell below the 12% non-inferiority margin. The trial is registered with ClinicalTrials.gov, NCT03959527, and EudraCT, 2019-000990-22.

**Findings:**

Between Nov 6, 2019, and March 16, 2023, 1011 patients were screened. 81 patients did not meet screening criteria and 930 participants were randomly assigned to zoliflodacin (n=621) or comparator (n=309). The mean participant age was 29·7 years (SD 9·4). 815 (88%) of 930 participants were assigned male at birth and 115 (12%) participants were assigned female at birth. 514 (55%) of 930 participants were Black or African American, 285 (31%) were Asian, and 113 (12%) were White. Microbiological cure rates at TOC in the microbiological intention-to-treat (urogenital) population (primary efficacy endpoint) were 460 (90·9%, 95% CI 88·1–93·3) of 506 participants for zoliflodacin and 229 (96·2%, 92·9–98·3) of 238 participants for comparator. The estimated difference between groups was 5·3% (95% CI 1·4–8·6) and the upper confidence interval limit was within the prespecified non-inferiority margin of less than 12%. Zoliflodacin was generally well tolerated and adverse events were similar between treatment groups. The most frequently reported treatment-emergent adverse events included headache (61 [10%] of 619 patients), neutropenia (42 [7%]), and leukopenia (24 [4%]) in the zoliflodacin group and injection site pain (38 [12%] of 308 patients), neutropenia (24 [8%]), and diarrhoea (22 [7%]) in the comparator group. The majority of adverse events were mild or moderate in severity. No serious adverse events were reported.

**Interpretation:**

Zoliflodacin was non-inferior to ceftriaxone plus azithromycin for the treatment of uncomplicated urogenital gonorrhoea and had a similar safety profile. These data suggest a potential role for zoliflodacin as an effective oral treatment option for uncomplicated urogenital gonorrhoea.

**Funding:**

German Federal Ministry of Research, Technology and Space, UK Department of Health and Social Care as part of the Global Antimicrobial Resistance Innovation Fund, Japan Ministry of Health, Labour and Welfare, Netherlands Ministry of Health, Welfare and Sport and Directorate-General for International Cooperation, Switzerland Federal Office of Public Health, and the Canton of Geneva, Switzerland.

## Introduction

In 2020, WHO estimated that *Neisseria gonorrhoeae* caused 82·4 million new cases of gonorrhoea among those aged 15–49 years worldwide.^1^ Without effective treatment, the morbidity associated with gonorrhoea complications can be considerable.^2^ Additionally, *N gonorrhoeae* infection enhances the risk of acquiring or transmitting HIV.^3^, ^4^

*N gonorrhoeae* has developed resistance to all current and previously used classes of antibiotics used to treat gonorrhoea, with ceftriaxone the sole remaining empirical treatment option for first-line gonorrhoea monotherapy.^5^ In 2017–18, the WHO Gonococcal Antimicrobial Surveillance Programme (GASP) reported high global rates of fluoroquinolone resistance, increasing azithromycin resistance, and, worryingly, progressively increasing multidrug resistance, including to extended-spectrum cephalosporins, such as ceftriaxone.^5^ Resistance patterns vary considerably by region; the WHO Enhanced GASP has highlighted situations of particular concern in Cambodia and Viet Nam. In 2022, nearly a third of isolates (32%) in Cambodia showed resistance to either broad-spectrum cephalosporins or azithromycin (with 7% showing resistance to both),^6^ and in Viet Nam, 27% of isolates were resistant to ceftriaxone in 2023.^7^ The WHO global action plan highlighted the development of new antibiotics for gonorrhoea as a high priority, and in 2019 the US Centers for Disease Control and Prevention declared antimicrobial-resistant *N gonorrhoeae* an urgent public health threat.^8^

Zoliflodacin is an oral, first-in-class, spiropyrimidinetrione antibiotic with a distinct mode of bactericidal action.^9^, ^10^ Zoliflodacin stabilises and arrests the cleaved covalent complex of DNA gyrase with double-stranded broken DNA and blocks ligation of this complex to form fused circular DNA.^9^ Zoliflodacin-resistant mutants generated in the laboratory indicate that the GyrB subunit of DNA gyrase is the primary target of zoliflodacin, unlike fluoroquinolones, which primarily interact with the GyrA subunit of DNA gyrase and the ParC subunit of topoisomerase IV.^11^, ^12^ Zoliflodacin maintains activity against ciprofloxacin-resistant, ceftriaxone-resistant, and azithromycin-resistant *N gonorrhoeae*,^12^ showing potent in-vitro antibacterial activity with a minimum inhibitory concentration for 90% of isolates of 0·125 μg/mL.^12^, ^13^ Phase 1 safety and pharmacokinetic studies, and pharmacokinetic and pharmacodynamic modelling, support a single oral 3 g treatment regimen, which was subsequently shown to be well tolerated and efficacious in treating uncomplicated urogenital gonorrhoea in a phase 2 study.^12^, ^14^, ^15^, ^16^, ^17^, ^18^ This oral formulation has the potential to substantially improve treatment access and thereby contribute to achieving the WHO goal of a 90% reduction in gonorrhoea infections by 2030.^19^

**Research in context**

**Evidence before this study**

Gonorrhoea represents a pressing global public health concern. In the past decade, sexually transmitted infections caused by *Neisseria gonorrhoeae* have steadily increased, with WHO estimating a global incidence of over 82 million cases per year in 2020. Effective antimicrobial treatment for gonorrhoea can help prevent transmission and long-term sequelae, but *N gonorrhoeae* has developed resistance to multiple first-line and second-line treatments. Zoliflodacin is a first-in-class spiropyrimidinetrione that inhibits bacterial DNA replication using a distinct mechanism of action and has a new target (GyrB), with potent in-vitro activity against *N gonorrhoeae* including multidrug-resistant strains. A phase 2 study found zoliflodacin efficacious in treating uncomplicated urogenital gonorrhoea, warranting further clinical investigation. This multinational, phase 3, randomised, controlled, non-inferiority clinical trial compared the efficacy and safety of a single oral dose of zoliflodacin 3 g with ceftriaxone 500 mg plus azithromycin 1 g for treatment of uncomplicated urogenital gonorrhoea. Before study conception and design, no formal literature search was undertaken, given the paucity of clinical development underway at the time. Instead, scientific advice was sought from global experts. Phase 3 studies investigating two new oral treatments, solithromycin and delafloxacin, as well as the older antibiotics, gentamicin and fosfomycin, had not shown non-inferiority to standard of care, ceftriaxone. However, ertapenem showed non-inferiority versus ceftriaxone for the treatment of anogenital *N gonorrhoeae* but had a higher frequency of adverse events and, being a broad spectrum antibiotic, concerns over stewardship and the need to reserve this antibiotic for difficult-to-treat serious bacterial infections is a limitation to its widespread use as a gonorrhoea treatment. Other than gepotidacin, which was entering phase 3 development concurrently, no new agents were identified in the development pipeline.

**Added value of this study**

This large study was done in a highly diverse population, including regions with a high burden of gonorrhoea. Zoliflodacin showed non-inferiority to the comparator for treating uncomplicated urogenital gonorrhoea and had a similar safety profile. Analysis of microbiological cure rate at pharyngeal and rectal sites of infection showed similar efficacy outcomes between the study treatment groups.

**Implications of all the available evidence**

Our findings provide evidence for zoliflodacin as a potential oral treatment for uncomplicated urogenital gonorrhoea.

Late-phase clinical development of zoliflodacin has been achieved through a unique public–private partnership between the Global Antimicrobial Research and Development Partnership (GARDP) Foundation (a global health-focused non-profit organisation) and Innoviva Speciality Therapeutics (a biotechnology company). GARDP sponsored and conducted a clinical trial to evaluate the use of zoliflodacin in patients with uncomplicated urogenital gonorrhoea.

## Methods

### Study design and participants

This phase 3, multinational, open-label, randomised, controlled, non-inferiority trial was designed and conducted in accordance with US Food and Drug Administration (FDA) guidance^20^ and advice from research and public health bodies. Community consultation was used to inform conduct of the trial, particularly recruitment. Some sites solicited input into the conduct of this trial through community working groups and advisory boards, representing Department of Health clinics, civil society, and the research sector. The study protocol and its amendments were approved by the institutional review board or ethics committee of all participating centres (appendix pp 4–8), and all participants provided written informed consent. The protocol and statistical analysis plan can be accessed online. Important changes to the protocol after the trial commenced were the inclusion of participants aged 12 years and older and the addition of exclusion criteria for CYP3A4 inhibitors and prohibition of concomitant CYP3A4 inhibitors (version 3.0); removal of *Chlamydia trachomatis* and *Mycoplasma genitalium* assessments for the day 30 visits and the addition of mandatory food intake before dosing (version 4.0); and increase of the prespecified non-inferiority margin to 12% for the primary endpoint with rationale and re-estimation of the evaluable patient population for the microbiological intention-to-treat population based on blinded pooled data analysis of study performance versus baseline assumptions (version 5.0). The trial is registered with ClinicalTrials.gov, NCT03959527, and EudraCT, 2019-000990-22.

The trial was done in 17 outpatient clinics in Belgium, the Netherlands, South Africa, Thailand, and the USA. Participating countries with high disease prevalence were identified for participation in the study. Sites selected for participation were led by principal investigators with research experience, who were knowledgeable in HIV and sexually transmitted infection and treatment. Feasibility questionnaires and prestudy visits assessed sexually transmitted infection case management guidelines, clinical services and resources (ie, facility, staff, proposed composition of the study team, standard sexually transmitted infection services offered at the site, assessment of laboratory capacity, research experience, and ethical review of clinical trials). Individuals aged 12 years and older with signs and symptoms consistent with urethral or endocervical gonorrhoea, a positive laboratory test confirming urogenital gonorrhoea in the preceding 14 days, or a history of unprotected sexual contact in the preceding 14 days with a partner confirmed to be infected with *N gonorrhoeae* were eligible for inclusion (see appendix pp 11–12 for full inclusion and exclusion criteria). Notable exclusion criteria included use of a systemic or intravaginal antibiotic with activity against *N gonorrhoeae* or using moderate or strong CYP3A4 inhibitors or inducers in the past 30 days, confirmed or suspected complicated or disseminated gonorrhoea, and pregnant or breastfeeding participants. People living with HIV were not excluded. Participants were recruited through outpatient sexually transmitted infection clinics. Demographic data about sex and gender were collected through self-reporting by study participants. Race and ethnicity were also self-reported.

### Randomisation and masking

Participants were randomly assigned (2:1) to receive a single dose of either zoliflodacin or comparator (ceftriaxone plus azithromycin). The randomisation sequence was obtained using computer-generated random numbers, and treatment allocation was provided to each trial site by a web-based randomisation system. Randomisation was done using random permutated blocks of three, six, and nine with stratification by assigned sex at birth; each block maintained the 2:1 randomisation ratio. The randomisation sequence was generated by the statistician from ICON Clinical Research (independent of the study statistician) and accessed via an interactive response technology platform. Following confirmation of participant eligibility, appropriately delegated and trained site personnel generated a treatment assignment report. This was used to communicate the assignment to personnel responsible for dispensing and administering the study treatments. The same personnel could be responsible for enrolment, randomisation, and dispensing and administering the study treatment (this would vary between sites).

The study was open-label with treatment assignment known to participants, clinical trial site personnel, and site-facing sponsor team members. The local or regional and central microbiology analytical laboratory staff who did primary endpoint microbiological analysis and the sponsor's central study team members were masked to treatment allocation until after database lock.

### Procedures

The study schedule of assessments is shown in the appendix (pp 9–10). Enrolled participants completed screening assessments on day 1 and, if eligible, were randomly assigned and received their allocated treatment on day 1. Eligible participants were randomly assigned before completion of all baseline laboratory assessments.

Participants who had not consumed a substantial meal within 2 h of dosing were provided with a standard moderate calorie and moderate fat meal, and assigned treatment was administered as soon as possible thereafter on day 1 (appendix pp 9–10): either a single oral dose of zoliflodacin 3 g (granules as oral suspension) or the comparator, a single intramuscular 500 mg dose of ceftriaxone plus a single oral 1 g dose of azithromycin, the dose regimen that was the standard treatment in the WHO guideline for gonorrhoea at the time the study was initiated.^21^ Appropriately trained and delegated study personnel delivered the intervention and comparator. Participants were instructed to abstain from sexual intercourse or use condoms for vaginal, anal, and oral sex to avoid re-infection for the duration of the study.

Appropriate clinical specimens (ie, urethral or endocervical, pharyngeal, or rectal swabs) were collected from all participants at baseline and at the test-of-cure (TOC) visit for Gram stain (baseline only), *N gonorrhoeae* culture with antimicrobial susceptibility testing (AST), and nucleic acid amplification test (NAAT) for *Chlamydia trachomatis* (baseline only) and *N gonorrhoeae*. Additional baseline assessments included safety blood tests, urine pregnancy tests, and HIV testing where applicable.

To maximise recovery of *N gonorrhoeae* from clinical specimens, collection and culture procedures followed WHO guidance.^22^ Local or regional microbiology laboratories performed culture for presumptive *N gonorrhoeae* identification. Presumptive *N gonorrhoeae* isolates were frozen and shipped to the central laboratory (JMI Laboratories; Iowa City, IA, USA) for definitive identification, using matrix-assisted laser desorption-ionisation time-of-flight mass spectrometry, and to determine minimum inhibitory concentrations (MICs) for zoliflodacin, azithromycin, cefixime, ceftriaxone, ciprofloxacin, gentamicin, tetracycline, and spectinomycin by the agar dilution method.^23^ Central laboratory results were considered definitive for the primary endpoint and other microbiological outcomes. Additional details on the microbiological and safety procedures are included in the appendix (pp 13–14).

Microbiology test results obtained from the local laboratory guided ongoing clinical management, including collection of post-baseline microbiological samples for individual participants (appendix p 15). Participants with an a posteriori diagnosis of *C trachomatis* infection by NAAT at baseline and who were randomly assigned to the zoliflodacin group were treated for *C trachomatis* infection as per local standard of care after completion of assessments at the TOC visit.

Participants attended post-baseline visits for safety assessments, including targeted physical examination, treatment-emergent adverse events, safety blood tests, pregnancy testing, collection of sexual history data, and microbiological swabs for culture and AST and NAAT (appendix pp 9–10). A pharmacokinetic sub-study was done and will be reported elsewhere.

### Outcomes

The primary objective was to assess the efficacy of a single oral 3 g dose of zoliflodacin compared with a combination of a single intramuscular 500 mg dose of ceftriaxone and a single oral 1 g dose of azithromycin. Assessment of safety and tolerability was included as a secondary endpoint. The primary efficacy assessment was done at the TOC visit on day 6 (± 2 days) and participants were followed up to day 30 (± 3 days).

Definitions of all study objectives and endpoints and analysis populations are provided in the appendix (pp 16–17). The primary endpoint was the proportion of participants with microbiological cure (negative or indeterminate *N gonorrhoeae* culture) at the urogenital site at TOC among participants included in the microbiological intention-to-treat (urogenital) population.

Secondary endpoints evaluated at TOC were the proportion of participants with microbiological cure as determined by culture at rectal and pharyngeal sites in the microbiological intention-to-treat (rectal and pharyngeal) populations, the proportion of male participants with clinical cure in the clinical cure population, the proportion of female and male participants with microbiological cure as determined by endocervical or urethral culture in the microbiological intention-to-treat (urogenital) population, the proportion of participants with microbiological cure as determined by urogenital culture and for whom the baseline AST profile indicated pre-existing resistance to antibiotics commonly used for gonorrhoea treatment (including to ceftriaxone and azithromycin), the AST profile of gonococcal isolates, and the proportion of participants with a negative *N gonorrhoeae* NAAT from urogenital, pharyngeal, and rectal sites. Definitions used for culture and NAAT result interpretation are available in the appendix (p 18). Primary and secondary microbiological endpoints (pathogen identification and susceptibility testing) were centrally assessed.

Adverse event data were collected systematically. All directly observed adverse events, adverse events spontaneously reported by the participant, and adverse events elicited through open-ended questioning of the participant or study assessments were recorded. Investigators graded adverse event severity according to the Common Terminology Criteria for Adverse Events (version 5.0).

### Statistical analysis

The microbiological intention-to-treat populations (urogenital, rectal, and pharyngeal) included all randomly assigned participants who had a positive *N gonorrhoeae* culture from the respective anatomical site at baseline and for which AST showed no pre-existing resistance to both ceftriaxone and azithromycin as confirmed by the central laboratory (appendix p 17).

The evaluable populations (urogenital, rectal, and pharyngeal) included those in the microbiological intention-to-treat populations who did not vomit within 30 min of administration of zoliflodacin or azithromycin and who had a *N gonorrheae* culture result at TOC.

Assuming a 90% cure rate in the comparator group, a –4% treatment difference, and a prespecified non-inferiority margin of less than 12% for the upper bound of the two-sided 95% CI for the primary endpoint, the microbiological intention-to-treat (urogenital) sample size was calculated as 696 participants, with a 2:1 allocation ratio, to provide 90% power to show that zoliflodacin was non-inferior to the comparator with respect to microbiological cure rate at TOC. The 2:1 randomisation ratio was chosen to allow sufficient safety data to be collected for zoliflodacin.^24^ Assuming up to 25% exclusion from the microbiological intention-to-treat (urogenital) population, 928 randomly assigned participants were needed to achieve the target sample size of 696.

For the primary endpoint analysis, the proportion of participants with microbiological cure at TOC was calculated for the microbiological intention-to-treat (urogenital) population, and 95% CIs were calculated using the Clopper–Pearson method. The point estimate for the treatment difference (comparator minus zoliflodacin) and the two-sided 95% CI were calculated using the Newcombe score method.^25^ Participants included in the microbiological intention-to-treat who had non-assessable outcomes at TOC (eg, missed or out-of-window TOC visits, or samples taken as per protocol but without results) were analysed as microbiological failures. Subgroup analyses by prespecified baseline characteristics were done in the same manner as the primary analysis.

Sensitivity analyses of the primary endpoint analysis were done in the evaluable (urogenital) population, with assessment of cure in participants with samples collected out-of-window of the TOC visit in the microbiological intention-to-treat population and imputation of missing data using predicted values from a multivariable logistic regression model that included these observed participant data adjusted for baseline covariates. Multiple Imputation methods using missing-at-random (MAR) and missing-not-at-random (MNAR) pattern mixture models were applied to create datasets with replacement of missing values of the primary endpoint. The missing data were imputed with predicted values from a multivariable logistic regression model that included these observed participant data adjusted for baseline covariates.

Safety and tolerability were assessed in the safety population (all randomly assigned participants who received any part of trial treatment). Treatment-emergent adverse events were summarised by incidence, severity, causality, and seriousness. Changes from baseline in safety laboratory test results and targeted physical examinations were also summarised. An independent data safety monitoring board monitored the safety of participants during the study.

Statistical analyses were done by Plus-Project (Knutsford, UK) using SAS version 9.4.

### Role of the funding source

The sponsor of the study, GARDP, was involved in the study design, conduct, analysis, and interpretation of data and ensuring study data integrity, including information provided in the manuscript. GARDP received funding from bodies, acknowledged at the end of this publication, to conduct this study. These funding bodies had no role in study design, data collection, data analysis, data interpretation, or writing of the report.

## Results

Between Nov 6, 2019, and March 16, 2023, 1011 participants were screened at 16 of the 17 sites. One site screened an additional 32 participants (31 of whom were randomly assigned and treated); however, after identification of a serious breach of Good Clinical Practice, all participant data from this site were excluded from statistical analyses. This exclusion did not affect the statistical power of the study or the efficacy analyses because sufficient evaluable participants were enrolled without including data from this centre.

**Figure 1:**
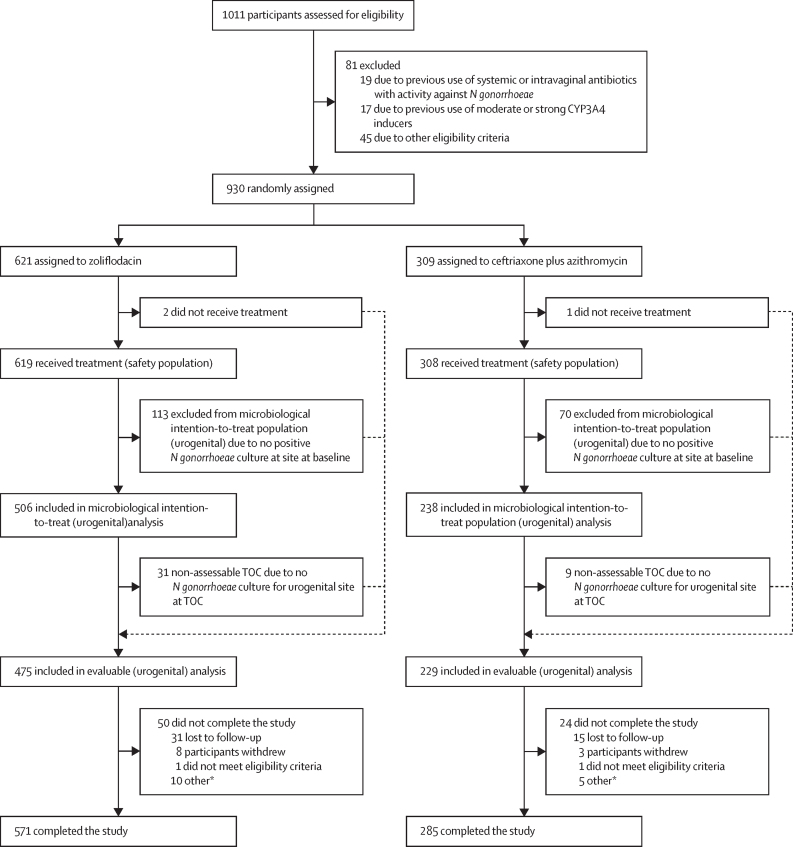
Trial profile *N gonorrhoeae*=*Neisseria gonorrhoeae*. TOC=test-of-cure. *Participants missed or were out of window for their end-of-trial visit.

81 patients did not meet screening criteria and 930 participants were randomly assigned to zoliflodacin (n=621) or comparator (n=309). 571 (92%) of 621 participants in the zoliflodacin group and 285 (92%) of 309 participants in the comparator group completed the study; loss to follow-up was the most common reason for not completing the study in both groups (figure 1). Among the 930 randomly assigned participants, three (<1%) did not receive study treatment, resulting in 927 (>99%) participants (619 in the zoliflodacin and 308 in the comparator group) comprising the safety population. 744 (80%) of 930 participants had a confirmed baseline urogenital *N gonorrhoeae* isolate for which AST showed no pre-existing resistance to both ceftriaxone and azithromycin and were therefore included in the microbiological intention-to-treat (urogenital) population. Reasons for exclusion from the analysis populations are summarised in the appendix (pp 19–21).

815 (88%) of 930 participants were assigned male at birth and 115 (12%) participants were assigned female at birth. 514 (55%) of 930 participants were Black or African American, 285 (31%) were Asian, and 113 (12%) were White. The mean participant age was 29·7 years (SD 9·4). Overall, 14 (2%) participants were younger than 18 years (range 15–17 years). Most participants were from South Africa (455 [46%] participants), followed by Thailand (270 [29%] participants), the USA (158 [17%] participants), and Europe (78 [8%] participants). Over half of participants in both groups reported a previous history of a sexually transmitted infection and 199 (21%) were living with HIV (table 1). Further baseline assessments and previous and concomitant medication use are detailed in the appendix (p 22). Baseline characteristics were similar between treatment groups (table 1; appendix p 22).

**Table 1:** Participant demographics and baseline characteristics

			**Zoliflodacin group (n =621)**	**Ceftriaxone plus azithromycin (n=309)**
**Randomly assigned population**				
Age, years				
	Mean (SD)		30·0 (9·6)	29·2 (9·1)
	Range		16–73	15–67
Sex assigned at birth				
	Male		544 (88%)	271 (88%)
	Female		77 (12%)	38 (12%)
Gender				
	Male		543 (87%)	269 (87%)
	Female		78 (13%)	40 (13%)
Race				
	Black or African American		349 (56%)	165 (53%)
	Asian		193 (31%)	92 (30%)
	White		66 (11%)	47 (15%)
	American Indian or Alaska Native		8 (1%)	1 (<1%)
	Native Hawaiian or Other Pacific Islander		2 (<1%)	1 (<1%)
	White, Asian		1 (<1%)	2 (1%)
	Other		2 (<1%)	1 (<1%)
Region				
	South Africa		278 (45%)	146 (47%)
	Thailand		181 (29%)	89 (29%)
	USA		107 (17%)	51 (17%)
	Europe		55 (9%)	23 (7%)
HIV status*				
	Negative		455 (73%)	234 (76%)
	Positive		134 (22%)	65 (21%)
	Missing		32 (5%)	10 (3%)
Fasted status†				
	Fed		555 (89%)	273 (88%)
	Fasted		64 (10%)	35 (11%)
	Missing		2 (<1%)	1 (<1%)
**Microbiological intention-to-treat population (urogenital)**				
Clinical assessment (urogenital)				
	Symptoms in males at birth			
		Urethral discharge	446/456 (98%)	216/220 (98%)
		Dysuria	392/456 (86%)	188/220 (85%)
	Symptoms in females at birth			
		Dysuria	17/50 (34%)	7/18 (39%)
		Abnormal vaginal discharge	36/50 (72%)	11/18 (61%)
		Postcoital bleeding	5/50 (10%)	0
		Vaginal bleeding between periods	9/50 (18%)	1/18 (6%)
		Vulvovaginal irritation	16/50 (32%)	7/18 (39%)

Data are n (%) or n/N (%), unless otherwise indicated.

* HIV positive status confirmed by positive test from sample or by other evidence which included medical history or indicated previous or concomitant medication.

† Fasted status: fed=food consumed ≤2 h before dosing; fasted=last meal >2 h before dosing.

The proportions of randomly assigned participants with confirmed positive *N gonorrhoeae* culture results were 744 (80%) of 930, 114 (12%) of 930 (12%), and 81 (9%) of 930 for the urogenital, rectal, and pharyngeal sites, respectively. Baseline microbiological assessments by study group and anatomical site for *N gonorrhoeae* culture and NAAT and *C trachomatis* and *Mycoplasma genitalium* NAAT are available in the appendix (p 23). Concordance between the local laboratory and central laboratory for identification of *N gonorrhoeae* isolates was more than 98% (appendix pp 24–25).

AST results for confirmed baseline *N gonorrhoeae* isolates are summarised by anatomical site in the appendix (pp 26–28). Zoliflodacin MIC values ranged from less than or equal to 0·008 μg/mL to 0·5 μg/mL. No baseline urogenital or rectal *N gonorrhoeae* isolates and one pharyngeal isolate were resistant to ceftriaxone (MIC >0·25 μg/mL) by Clinical and Laboratory Standards Institute interpretation criteria.^23^ Resistance of baseline isolates to azithromycin (6–11%), ciprofloxacin (75–86%), and tetracycline (92–100%) was consistent across anatomical sites. One urogenital, one pharyngeal, and two rectal isolates were resistant to ceftriaxone (MIC >0·125 μg/mL) by European Committee on Antimicrobial Susceptibility Testing breakpoints.^26^

Overall, *C trachomatis* co-infections at urogenital, rectal, and pharyngeal sites were detected by NAAT for 235 (25%) of 923 participants, 104 (11%) of 922 participants, and 28 (3%) of 925 participants, respectively.

Microbiological cure rates at TOC in the microbiological intention-to-treat (urogenital) population (primary efficacy endpoint) were 460 (90·9%, 95% CI 88·1–93·3) of 506 participants for zoliflodacin and 229 (96·2%, 92·9–98·3) of 238 participants for comparator. The estimated difference between groups (comparator minus zoliflodacin) was 5·3% (95% CI 1·4–8·6) and the upper confidence interval limit was within the prespecified non-inferiority margin of less than 12% (table 2). Among participants in the microbiological intention-to-treat (urogenital) population with microbiological failure at TOC (zoliflodacin 46 [9%] of 506 participants; comparator nine [4%] of 238 participants), 15 (3%) participants in the zoliflodacin group and none in the comparator group had confirmed *N gonorrhoeae* isolated at TOC. The remaining participants had non-assessable outcomes at TOC (ie, missed or out-of-window TOC visits, samples taken as per protocol but without results; 31 [6%] 506 participants in the zoliflodacin group and nine [4%] of 238 participants in the comparator group).

**Table 2:** Microbiological cure rate at TOC at urogenital site

		**Zoliflodacin**		**Ceftriaxone plus azithromycin**		**Difference (95% CI)**
		n/N	Proportion with microbiological cure (95% CI)	n/N	Proportion with microbiological cure (95% CI)	
**Primary efficacy endpoint**						
Microbiological intention-to-treat*		460/506	90·9% (88·1–93·3)	229/238	96·2% (92·9–98·3)	5·3% (1·4–8·6)
**Sensitivity analysis**						
Evaluable		460/475	96·8% (94·8–98·2)	229/229	100% (98·4–100)	3·2% (1·1–5·1)
Microbiological intention-to-treat including out-of-window assessments		471/506	93·1% (90·5–95·1)	231/238	97·1% (94·0–98·8)	4·0% (0·4–6·9)
Microbiological intention-to-treat excluding key protocol deviations		456/502	90·8% (88·0–93·2)	227/236	96·2% (92·9–98·2)	5·3% (1·4–8·7)
Microbiological intention-to-treat with multiple imputation						
	Missing at random†	NA	NA	NA	NA	3·1% (1·3–4·8)
	Missing not at random†	NA	NA	NA	NA	3·2% (1·4–5·0)
**Secondary analysis**						
Per-protocol‡		434/452	96·0% (93·8–97·6)	218/219	99·5% (97·5–100)	3·5% (1·0–5·8)

NA=not applicable. TOC=test of cure.

* The microbiological intention-to-treat population and modified microbiological intention-to-treat population were identical, therefore the secondary analysis of the modified microbiological intention-to-treat population is not shown.

† Multiple imputation analysis generated multiple instances of the dataset using a logistic regression model, and the results were pooled to produce a unique point estimate of the risk difference; cure rates in each individual treatment group were not estimated.

‡ All participants in the microbiological intention-to-treat population who met all inclusion (and did not meet exclusion) criteria; complied with trial treatment; did not vomit within 30 min of administration of zoliflodacin or azithromycin; did not receive any systemic antibiotic with known activity against *Neisseria gonorrhoeae* before the TOC visit; did not receive any of the prohibited medications; abstained from sexual intercourse or used condoms for vaginal, anal, and oral sex before TOC; and returned to the trial site for the TOC visit within the specified window.

In an analysis of the primary efficacy endpoint using the more clinically relevant evaluable population, which excluded participants with non-assessable outcomes at TOC, the urogenital microbiological cure rates were 460 (96·8%, 95% CI 94·8–98·2) of 475 participants for the zoliflodacin group and 229 (100%, 98·4–100) of 229 participants for the comparator group, a difference of 3·2% (95% CI 1·1–5·1). Sensitivity analyses of the primary endpoint in all other analysis populations showed similar, high microbiological cure rates for both treatment groups, supporting the overall findings of the primary analysis (table 2; appendix p 29). Further sensitivity analyses in the microbiological intention-to-treat population with multiple imputation for missing data (MAR and MNAR) also showed similar outcomes, as might be expected considering the small amount of missing data, indicating a robust primary endpoint analysis.

Microbiological cure rates at TOC for both the pharyngeal and rectal sites (secondary endpoints; microbiological intention-to-treat population) were similar between treatment groups (table 3). In the microbiological intention-to-treat population (pharyngeal), cure rates were 42 (79·2%, 95% CI 65·9–89·2) of 53 participants in the zoliflodacin group and 22 (78·6%, 59·0–91·7) of 28 participants in the comparator group. In the microbiological intention-to-treat population (rectal), cure rates were 69 (87·3%, 78·0–93·8) of 79 participants in the zoliflodacin group and 31 (88·6%, 73·3–96·8) of 35 participants in the comparator group. In the evaluable populations (pharyngeal and rectal), cure rates exceeded 90% in both treatment groups (table 3).

**Table 3:** Microbiological cure rate at TOC at pharyngeal and rectal sites (secondary endpoints)

	**Zoliflodacin**		**Ceftriaxone plus azithromycin**		**Difference (95% CI)**
	n/N	Proportion with microbiological cure (95% CI)	n/N	Proportion with microbiological cure (95% CI)	
**Pharyngeal**					
Microbiological intention-to-treat*	42/53	79·2% (65·9 to 89·2)	22/28	78·6% (59·0 to 91·7)	−0·7% (−20·8 to 16·3)
Evaluable	42/46	91·3% (79·2 to 97·6)	22/23	95·7% (78·1 to 99·9)	4·3% (−13·1 to 16·5)
Per-protocol†	39/46	84·8% (71·1 to 93·7)	20/23	87·0% (66·4 to 97·2)	2·2% (−18·4 to 17·7)
**Rectal**					
Microbiological intention-to-treat*	69/79	87·3% (78·0 to 93·8)	31/35	88·6% (73·3 to 96·8)	1·2% (−14·3 to 12·6)
Evaluable	69/72	95·8% (88·3 to 99·1)	31/31	100% (88·8 to 100)	4·2% (−7·2 to 11·5)
Per-protocol†	61/64	95·3% (86·9 to 99·0)	30/31	96·8% (83·3 to 99·9)	1·5% (−11·9 to 10·1)

TOC=test of cure.

* The microbiological intention-to-treat population and modified microbiological intention-to-treat population were identical, therefore the secondary analysis of the modified microbiological intention-to-treat population is not shown.

† All participants in the microbiological intention-to-treat population who met all inclusion (and did not meet exclusion) criteria; complied with trial treatment; did not vomit within 30 min of administration of zoliflodacin or azithromycin; did not receive any systemic antibiotic with known activity against *Neisseria gonorrhoeae* before the TOC visit; did not receive any of the prohibited medications; abstained from sexual intercourse or used condoms for vaginal, anal, and oral sex before TOC; and returned to the trial site for the TOC visit within the specified window.

In a further secondary analysis, the clinical cure rate at TOC in participants assigned male at birth and with at least one sign or symptom of urethral gonorrhoea at baseline was similar for zoliflodacin (375 [82%] of 460 participants) and the comparator (194 [88%] of 220 participants), with a difference of 6·7% (95% CI 0·7–11·9; clinical cure population; appendix p 30).

**Figure 2:**
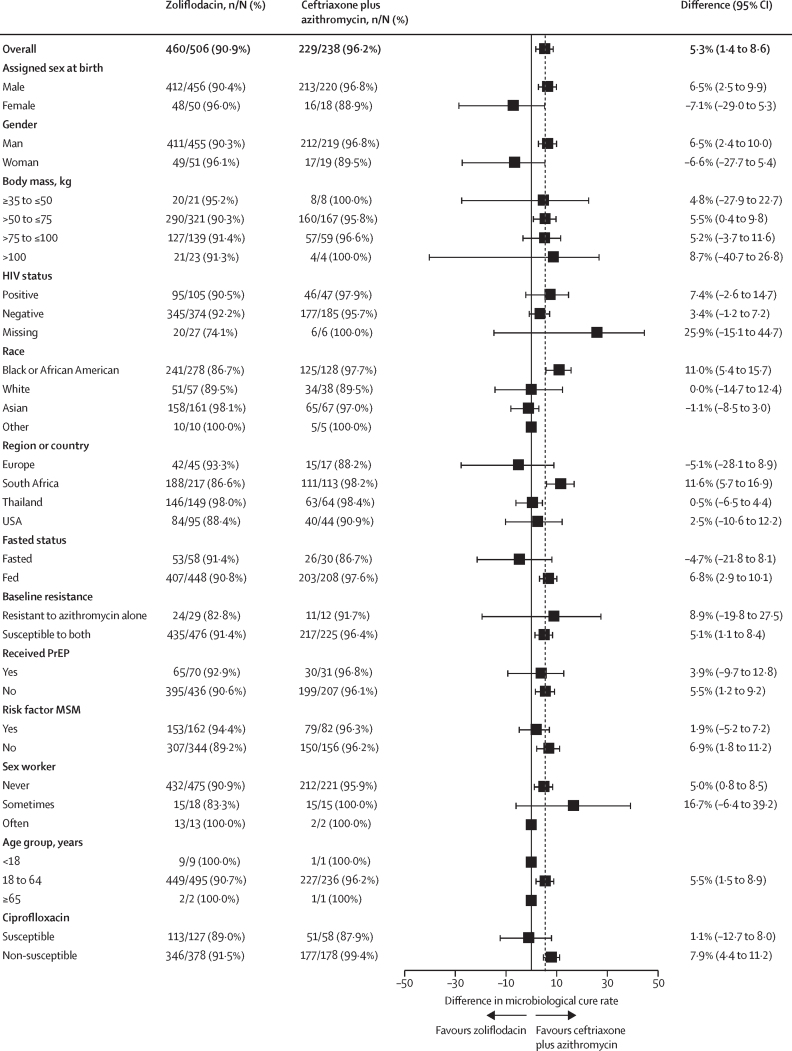
Forest plot of difference in microbiological cure rate at test of cure by subgroup: microbiological intention-to-treat (urogenital) population The dashed vertical line indicates the overall treatment difference. MSM=men who have sex with men. PrEP=pre-exposure prophylaxis.

Planned subgroup analyses of the primary endpoint are shown in figure 2 and the appendix (pp 31–33). In general, the differences in urogenital microbiological cure rate at TOC for zoliflodacin and comparator were consistent across subgroups. Some subgroups contained very low numbers of participants, precluding meaningful interpretation. All ten adolescent participants (aged 15–17 years, nine in the zoliflodacin group and one in the comparator group) in the microbiological intention-to-treat (urogenital) population had microbiological cure. Some numerical differences between treatment groups in microbiological cure rate at the urogenital site were observed for male and female subgroups, but overlapping CIs indicated a similar treatment effect overall. For participants with baseline *N gonorrhoeae* isolates resistant to azithromycin, microbiological cure rates were 24 (82·8%, 95% CI 64·2–94·2) of 29 participants for the zoliflodacin group and 11 (91·7%, 61·5–99·8) of 12 participants in the comparator group.

For all three anatomical sites, there were no notable shifts in zoliflodacin MICs of *N gonorrhoeae* isolates from baseline to TOC observed in either treatment group, indicating no evidence of emergence of resistance to zoliflodacin (appendix pp 34–36). A separate paper reporting clinical and microbiological data of participants with microbiological failure and exploratory results of whole-genome sequencing of the paired baseline and TOC isolates is in preparation. Eradication of *N gonorrhoeae* at day 6 (± 2 days) assessed by NAAT (appendix p 37) was similar between treatment groups.

Both zoliflodacin and the comparator were generally well tolerated and treatment-emergent adverse events were similar between treatment groups (table 4). No deaths or other serious treatment-emergent adverse events were reported. 286 (46%) of 619 participants in the zoliflodacin group and 143 (46%) of 308 participants in the comparator group reported at least one treatment-emergent adverse event. Most treatment-emergent adverse events were mild or moderate in severity (grade 1 or 2) in both treatment groups. The most frequently reported treatment-emergent adverse events included headache (61 [10%] participants), neutropenia (42 [7%] participants), and leukopenia (24 [4%] participants) in the zoliflodacin group and injection site pain (38 [12%] participants), neutropenia (24 [8%] participants), and diarrhoea (22 [7%] participants) in the comparator group. One grade 4 treatment-emergent adverse event was reported in the zoliflodacin group (neutrophil count decreased), but was asymptomatic and assessed by the investigator as unrelated to treatment. No other abnormal safety laboratory values or physical examination findings indicated safety concerns.

**Table 4:** Treatment-emergent adverse events in the safety population

		**Zoliflodacin group (N=619)**	**Ceftriaxone plus azithromycin group (N=308)**
Participants with at least one treatment-emergent adverse event		286 (46%); 450	143 (46%); 257
	Related treatment-emergent adverse events	117 (19%); 149	76 (25%); 113
	Serious treatment-emergent adverse events	0	0
	Related serious treatment-emergent adverse events	0	0
	Treatment-emergent adverse events leading to treatment discontinuation	0	0
	Treatment-emergent adverse events leading to death	0	0
Treatment-emergent adverse events by maximum severity			
	Grade 1—mild	156 (25%); 288	82 (27%); 167
	Grade 2—moderate	109 (18%); 140	43 (14%); 71
	Grade 3—severe	20 (3%); 21	18 (6%); 19
	Grade 4—life-threatening	1 (<1%); 1	0
	Grade 5—death	0	0
Treatment-emergent adverse events by preferred term (≥3% either group)			
	Headache	61 (10%); 65	14 (5%); 15
	Neutropenia	42 (7%); 42	24 (8%); 24
	Leukopenia	24 (4%); 24	7 (2%); 7
	Neutrophil count decreased	21 (3%); 21	15 (5%); 15
	Dizziness	21 (3%); 21	5 (2%); 5
	Nausea	16 (3%); 16	12 (4%); 13
	Diarrhoea	15 (2%); 15	22 (7%); 22
	Injection site pain	5 (1%); 6	38 (12%); 38

Data are the number of participants with at least one event in each group (%); number of events.

Both mean white blood cell and absolute neutrophil counts decreased from baseline to TOC, consistent with successful treatment of an acute infection (data not shown). At each timepoint, these parameters were consistently lower among Black or African American participants than among Asian, White, or other participants, with no difference observed between treatment groups (appendix p 38). Post-hoc multivariate logistic regression analysis revealed a strong association of neutropenia with race (Black or African American odds ratio 109·70, 90% CI 20·91–575·53; p<0·0001) and male sex (0·24, 0·11–0·53, p=0·0029) but not with other variables, including treatment group, HIV status, and concomitant use of antiretrovirals.

In adolescents, three treatment-emergent adverse events (one mild event of vulvovaginal candidiasis, one mild event of increased conjugated bilirubin, and one moderate event of increased blood bilirubin) were reported by two (17%) of 12 participants in the zoliflodacin group, and two treatment-emergent adverse events (one mild event of neutropenia and one moderate event of dizziness) were reported by two (100%) of two participants in the comparator group.

## Discussion

This phase 3, randomised, controlled clinical trial met its primary objective, showing that zoliflodacin (single oral dose of 3 g) was non-inferior to the comparator combination of ceftriaxone and azithromycin for the treatment of uncomplicated urogenital gonorrhoea. Consistent results were seen in sensitivity and subgroup analyses, supporting a robust primary endpoint analysis. Key secondary endpoint analyses of pharyngeal and rectal sites of infection showed similar rates of microbiological cure between treatment groups, although the study was not powered to show non-inferiority for these endpoints and the number of participants with extragenital infections was small despite a large study population. Considering the urgent global need for new gonorrhoea treatments, the finding that a single-dose of oral zoliflodacin was efficacious at curing urogenital and rectal and pharyngeal gonorrhoea is encouraging in the context of population-level public health efforts to control disease.

This finding is of particular importance given previous failures of potential oral treatments for uncomplicated urogenital gonorrhoea; neither solithromycin^27^ nor delafloxacin^28^ were efficacious alternatives to ceftriaxone in phase 3 non-inferiority studies. Older antibiotics, including gentamicin and fosfomycin, also did not show non-inferiority to ceftriaxone.^28^, ^29^ Ertapenem showed non-inferiority to ceftriaxone for anogenital *N gonorrhoeae* infections but had a higher adverse event frequency and, being a broad-spectrum antibiotic, concerns over stewardship and the need to reserve this antibiotic for difficult-to-treat serious bacterial infections is a limitation to its widespread use as a treatment for gonorrhoea.^30^ Gepotidacin was found to be non-inferior to the standard of care comparator for treatment of uncomplicated urogenital gonorrhoea and might represent another potential oral treatment option.^31^

In accordance with FDA guidance,^20^ we predefined a non-inferiority margin of 12% considering the paucity of effective treatment options to address the growing urgent public health need of multidrug-resistant gonorrhoea. Although the primary endpoint difference between treatment groups numerically favoured the comparator (as the lower limit of the 95% CI was >0%), the upper limit of the 95% CI was well within the 12% non-inferiority margin and indeed within a 10% margin. The primary endpoint analysis used the microbiological intention-to-treat population, wherein non-assessable outcomes at TOC (ie, missed or out-of-window TOC visits, samples taken per protocol but without results) were evaluated as microbiological failures. In the evaluable population, which excluded participants without an assessable TOC outcome, the difference in microbiological cure was in favour of the comparator, again with the upper limit of the CI well within the predefined non-inferiority margin. Analysis of the evaluable population—a more clinically relevant population representative of participants with a confirmed microbiological assessment at TOC—is generally used to inform clinical treatment guidance.^32^ The absence of urogenital microbiological failures in the comparator group (compared with 15 in the zoliflodacin group) might be partly attributable to the long half-life of azithromycin, potentially conferring prolonged prophylaxis against re-infection before TOC.

Single in-vitro mutations due to amino acid alterations in GyrB D429 or K450 have been associated with increased zoliflodacin MICs. A single GyrB Ser467Asn substitution, as a first step that can predispose strains to develop higher zoliflodacin MICs, or as a second step mutation, has also contributed to increased zoliflodacin MICs.^33^, ^34^, ^35^ Additional insights into microbiological failures from whole-genome sequencing analysis of paired baseline and TOC isolates, done as an exploratory objective, are presented in a separate manuscript currently in preparation. Given there were no meaningful shifts from baseline in zoliflodacin MICs among *N gonorrhoeae* isolates obtained at TOC, this sequencing is evidence for a lack of development of resistance during zoliflodacin treatment in this study.

The increasing spread of resistance to current treatments, including ceftriaxone and azithromycin, poses an urgent threat to global public health and has reinforced the need for novel therapeutic options to ensure that gonorrhoea remains treatable.^6^, ^36^ Additional benefits of this oral, single-dose regimen potentially include improved access to treatment for populations and individual patients who might otherwise go untreated (eg, by facilitating community-led care, access in remote locations, and self-administration or by expediting partner therapy), treatment of patients with β-lactam hypersensitivity or needle aversion, and use in regions where ceftriaxone resistance is prevalent. Such treatment options that remove barriers to receiving effective care, thereby reducing transmission, can have a broader positive impact on public health. Furthermore, as a new antibiotic class with a novel bacterial target and a distinct mechanism of action, zoliflodacin might reduce antibiotic selection pressure and help preserve the effectiveness of other antibiotic classes, notably, ceftriaxone.

The safety profiles of zoliflodacin and comparator treatment were similar. Incidence rates of gastrointestinal treatment-emergent adverse events with zoliflodacin and with comparator were generally consistent with oral antibiotic therapy, and no serious adverse events were reported in either group. Asymptomatic neutropenia was reported in more than 5% of participants in both groups and decreases in white blood cell and absolute neutrophil counts were strongly associated with being Black or African American and male. In the absence of clinical sequelae associated with low neutrophil count in the study, this observation is consistent with the reported phenomenon of benign constitutional neutropenia, which affects several ethnic groups, including 25–50% of individuals of African descent, and is a limitation to using standard White-based reference ranges and thresholds for classification of haematological abnormalities in a majority Black or African American study population.^37^

A major strength of this study is its size; this is the largest trial conducted to date in participants with *N gonorrhoeae* infection. Spanning four continents, the study was ethnically diverse and included a large representation from regions with a high burden of gonorrhoea in low-income and middle-income countries. The study population also included women and people living with HIV, both groups for whom effective gonorrhoea treatment is essential. In interpreting the study results, it is important to note that the choice of the dual-therapy comparator group was reflective of the standard of care at most study sites at the start of the study. Addition of azithromycin to ceftriaxone is no longer recommended for first-line treatment of uncomplicated gonorrhoea in many settings.^38^, ^39^

Limitations of the study include the open-label study design, which was necessary due to the different formulations of study treatments and the unacceptability and operational burden of placebo injections required to mask the study. The impact of this design aspect cannot be fully elucidated, but it could be postulated as a factor, for example, in the slight imbalance between treatment groups in missed TOC visits. Despite substantial community outreach measures implemented to enrich the population of women and adolescents in this study and waivers for parental consent where permitted, the numbers of individuals from these groups were still low. This result reflects the challenges that remain for enrolment of women and adolescents into gonorrhoea clinical trials—eg, the high rate of asymptomatic infection in women; eligibility criteria addressing contraception use, pregnancy, and breastfeeding; and heath-care seeking sensitivities among adolescents.

The efficacy of zoliflodacin in priority populations is an important area for future investigation, as is further exploration of use in extragenital infection, especially pharyngeal infection. Drug–drug interaction studies with anti-infectives frequently co-administered—eg, in syndromic management of sexually-transmitted infections and globally relevant endemic infections—should be considered.

Antibiotic product development is challenging and offers a low return on investment; many large pharmaceutical companies with previously active antibiotic pipelines are leaving the market. This trend coincides with a critical increase in antimicrobial resistance and an urgent global need for new effective treatments for priority pathogens. The GARDP–Innoviva Specialty Therapeutics product development partnership is an example of an innovative operating model that might successfully overcome some of the barriers to antibiotic development.

In conclusion, this trial showed the efficacy of a single dose of oral zoliflodacin for treatment of uncomplicated urogenital gonorrhoea. Zoliflodacin also showed similar efficacy outcomes to the comparator for rectal and pharyngeal infections and was well tolerated with a favourable safety profile. These data suggest a potential role for zoliflodacin as an effective oral treatment option for uncomplicated urogenital gonorrhoea.

### Zoliflodacin Phase 3 Study Group

### Contributors

### Data sharing

The data underlying the results of this study and related study documents (eg, study protocol, statistical analysis plan, and informed consent form) can be made available upon request. Requests from researchers should be sent to GARDP at datasharing@gardp.org for consideration. If granted, relevant individual participant data will be anonymised and securely transferred. Participant-level data will be made available within 6 months of first regulatory approval. A signed data sharing agreement might be required.

## Declaration of interests

The institutions or affiliated institutions of LAB, TAB, SEC, SD-M, HJCdV, JAD, KG, CK, RK, VN, NP, ES, SNT, and ZZ received funding from GARDP for the trial. Additionally, LAB has received investigator-initiated research support from Hologic (donation of test kits) and SpeeDx for trials unrelated to this study. SNT received institutional research funding from GlaxoSmithKline, Entasis Therapeutics, and Innoviva Specialty Therapeutics. NP has received research funding to their institution from Gilead Sciences and ViiV Healthcare. SEC has received test kits from Cepheid and Hologic for trials unrelated to this study. AL, SO, and MB are employees of GARDP. SS is a former employee of Entasis Therapeutics and of GARDP. HB is an employee of Plus-Project, consulting for GARDP. DL is an employee of Innoviva Specialty Therapeutics and owns stock. JPM is a former employee of Entasis-Innoviva Specialty Therapeutics. JPO is a former employee of Entasis-Innoviva Specialty Therapeutics and owns stock. EWH has received consulting fees from GARDP and Visby Medical. All other authors report no competing interests.
