# Supplementary material for: Zoliflodacin versus ceftriaxone plus azithromycin for treatment of uncomplicated urogenital gonorrhoea: an international, randomised, controlled, open-label, phase 3, non-inferiority clinical trial
Source: Lancet. 2026 Jan 10;407(10524):147–60. doi: 10.1016/S0140-6736(25)01953-1 (PMC12784215; doi:10.1016/S0140-6736(25)01953-1)
Supplement: Supplementary appendix [file mmc1.pdf]

THE LANCET

Supplementary appendix

This appendix formed part of the original submission and has been peer reviewed. We post it as supplied by the authors.

Supplement to: Luckey A, Balasegaram M, Barbee LA, et al. Zoliflodacin versus ceftriaxone plus azithromycin for treatment of uncomplicated urogenital gonorrhoea: an international, randomised, controlled, open-label, phase 3, non-inferiority clinical trial. *Lancet* 2025; published online Dec 11. https://doi.org/10.1016/S0140-6736(25)01953-1.

Supplementary Appendix

Zoliflodacin versus ceftriaxone plus azithromycin for the treatment of uncomplicated urogenital gonorrhoea: an international, randomised, controlled, open-label, phase 3, non-inferiority clinical trial

Table of contents

Supplementary Methods 4

Independent Ethics Committee or Institutional Review Board (IEC/IRB) approval details for participating study sites 4

Protocol amendments 6

Changes in the Conduct of the Study (Protocol Amendments) 6

Additional information on CSP amendments 6

Schedule of assessments 9

Inclusion and exclusion criteria 11

Inclusion criteria 11

Exclusion criteria 11

Microbiological procedures and assessments 13

Safety assessments 14

Use of microbiological results for subsequent clinical management 15

Objectives and endpoints 16

Analysis sets 17

Definitions used for microbiological assessments and nucleic acid amplification tes*ts* 18

Supplementary Results 19

Table S1. Analysis datasets summary 19

Baseline characteristics 22

Table S2. Additional participant demographics and baseline characteristics: randomised population 22

Prior and concomitant treatments 22

Baseline microbiological assessments 23

Table S3. Summary of microbiological evaluations by local laboratories: randomised population 23

Table S4. Comparison of N gonorrhoeae culture data from local laboratory vs central laboratory: randomised population 24

Baseline MIC distributions **26**

Table S5. Summary of MIC and antimicrobial susceptibility status for N gonorrhoeae at baseline: micro-ITT (urogenital) population 26

Table S6. Summary of MIC and antimicrobial susceptibility status for N gonorrhoeae at baseline: micro-ITT (pharyngeal) population 27

Table S7. Summary of MIC and antimicrobial susceptibility status for N gonorrhoeae at baseline: micro-ITT (rectal) population 28

Secondary endpoints **29**

Figure S2. Difference in microbiological cure rate at TOC (urogenital site including sensitivity analysis populations) 29

Table S8. Clinical cure rate at TOC in male-at-birth participants 30

Table S9. Microbiological cure rate at TOC at urogenital site in subgroups: micro-ITT and evaluable (urogenital) population 31

Table S10. Summary of MIC and antimicrobial susceptibility status for N gonorrhoeae at TOC: micro-ITT (urogenital) population 34

Table S11. Summary of MIC and antimicrobial susceptibility status for N gonorrhoeae at TOC: micro-ITT (pharyngeal) population 35

Table S12. Summary of MIC and antimicrobial susceptibility status for N gonorrhoeae at TOC: micro-ITT (rectal) population 36

Table S13. N gonorrhoeae NAAT results at TOC (all populations and anatomical sites) 37

Table S14. Summary of absolute neutrophil count by visit and race: safety population 38

Zoliflodacin Phase 3 Study Group .... Error! Bookmark not defined.

References **39**

Supplementary Methods

Independent Ethics Committee or Institutional Review Board (IEC/IRB) approval details for participating study sites

<table>
<tr><th>IEC/IRB name, address and study tracking number</th><th>Site</th></tr>
<tr><td colspan="2">USA</td></tr>
<tr><td rowspan="2">WCG IRB
WCG - North America
212 Carnegie Center, Suite 301,
Princeton, NJ 08540, USA

IRB tracking number: 20191331</td><td>• University of Alabama at Birmingham, Birmingham, AL
• Jefferson County Department of Health, Birmingham, AL
• Bell Flower Clinic, Indianapolis, IN
• San Francisco Department of Public Health City Clinic, San Francisco, CA</td></tr>
<tr><td>• Seattle and King County STD Clinic, Seattle, WA</td></tr>
<tr><td>LSUHSC Institutional Review Board
433 Bolivar Street,
Room 206,
New Orleans, LA 70112, USA

IRB number: 19-149</td><td>• Louisiana State University Health Sciences Center Public Health, New Orleans, LA</td></tr>
<tr><td colspan="2">Europe</td></tr>
<tr><td>UZ Antwerpen Ethisch comité UZA/UA
Drie Eikenstraat 655,
2650, Edegem, Belgium

Ref: Project ID 1879</td><td rowspan="2">• Institute of Tropical Medicine, Antwerp, Belgium</td></tr>
<tr><td>Institute of Tropical Medicine (ITM) Antwerp Institutional Review Board
Nationalestraat 155,
2000, Antwerp, Belgium

EC ref: 1549/21</td></tr>
<tr><td>METC Amsterdam UMC
AMC, (Room: TK0-270),
Meibergdreef 9, 1105 AZ, Amsterdam

NL number: NL70051.018.19
METC number: 2019_122</td><td>• Public Health Service (GGD) STI Outpatient Clinic, Amsterdam, Netherlands</td></tr>
<tr><td colspan="2">Thailand</td></tr>
<tr><td>Central Research Ethics Committee (CREC)
5th Fl Building2, The National Research Council of Thailand,196 Moo 5,
Phahonyothin Road,
Latyao, Chatuchak, Bangkok, 10900, Thailand

CREC number: CREC 057/62BPm</td><td>• Institute of HIV Research and Innovation (IHRI), Bangkok
• Silom Community Clinic, Bangkok</td></tr>
<tr><td>The Ethical Review Committee for Research in Human Subjects Ministry of Public Health (ECMOPH)
3rd Floor, Department of Medical Services Building,
Ministry of Public Health,
Tiwanon Road,
TaladKhwan Sub-district,
Muang District, Nonthaburi, 11000, Thailand

Ref no: 27/2562</td><td>• Silom Community Clinic, Bangkok
• Bangrak STI Center, Bangkok</td></tr>
<tr><td>Institutional Review Board Faculty of Medicine, Chulalongkorn University (CHULA MED IRB)
Anandamahidol Building 3rd fl.,
Faculty of Medicine,
Chulalongkorn University,
1873 Rama 4 Rd.,
Pathumwan, Bangkok, 10330, Thailand

IRB no: 183/63</td><td>• Institute of HIV Research and Innovation (IHRI), Bangkok</td></tr>
<tr><td>Ethics Committee of the Faculty of Tropical Medicine, Mahidol University (TMEC),
420/6 Ratchawithi Road,
Ratchathewi, Bangkok, 10400, Thailand

Ref no: CREC 057/62BPm</td><td>• Silom Community Clinic, Bangkok</td></tr>
<tr><td colspan="2">South Africa</td></tr>
</table>

IEC/IRB name, address and study tracking number	Site
**South African Medical Research (SAMRC) Human Research Ethics Committee (HREC)**<br>Francie van Zijl Drive<br>Parowvallei, Cape Town, 7505, South Africa<br><br>Protocol ID: EC012-8/2019	• SAMRC Botha's Hill Clinical Research Site, Botha's Hill<br>• SAMRC Tongaat Clinical Research Site, Tongaat
**Wits Human Research Ethics Committee (Medical)**<br>University of Witwatersrand,<br>Ethics Office 3rd Floor,<br>31 Princess of Wales Terrace,<br>Parktown, Johannesburg, 2193, South Africa<br><br>Ethics reference number: 190613B	• Wits RHI, University of the Witwatersrand, Johannesburg
**University of Cape Town Human Research Ethics Committee**<br>University of Cape Town,<br>E53.46 Old Main building,<br>Groote Schuur Hospital,<br>Observatory, 7925, South Africa<br><br>Reference number: 550/2021	• Masiphumelele Research Site, Cape Town
**Pharma-Ethics Independent Research Ethics Committee**<br>123 Amcor Road,<br>Lyttelton Manor, 0157, South Africa<br><br>Ethics reference no: 210824237	• Setshaba Research Centre, Soshanguve

Protocol amendments

Changes in the Conduct of the Study (Protocol Amendments)

Changes in the conduct of the study before the analysis was performed (including administrative changes) were incorporated into revised versions of the Clinical Study Protocol (CSP) as protocol amendments, as follows:

- Collection of date/time of last meal prior to dosing was to be recorded for all participants.
- Participants who were taking strong or moderate CYP3A4 inhibitors were excluded from the study.
- Contingency measures due to the COVID-19 pandemic:
    - Deletion of *Chlamydia trachomatis* assessment at Day 30 visit (exploratory endpoint).
    - Deletion of *Mycoplasma genitalium* assessments at all applicable visits (exploratory endpoint).
    - Clarification on handling of any out-of-window visits.
    - Change to allow different suppliers for the NAAT testing platform.
- Addition of mandatory food intake before dosing.
- Addition of new analysis population (Evaluable Population) and additional sensitivity analysis.
- Widening of the pre-specified non-inferiority margin for the primary endpoint from 10% to 12% and subsequent re-estimation of evaluable Micro-ITT population based on a blinded pooled data analysis of study performance versus baseline assumptions.

Additional information on CSP amendments

Food intake

The absorption of zoliflodacin and tolerability of azithromycin tablets are improved when participants are dosed in the fed state. Therefore, following a protocol amendment made early in the study, it was a requirement that food was taken before administration of trial treatment. If a participant had not eaten a substantial meal before assessment, they were to be provided with a meal as follows:

- ≤ 75 kg body weight: Within the 2 hours before dosing, the participant was to eat a meal with approximately 400 Kcal and 30% fat content
- > 75 kg body weight: Within the hour before dosing, the participant was to eat a meal with approximately 500 Kcal and 30% fat content

The date and time of food intake prior to trial treatment administration was to be recorded.

Participants received randomised treatment as soon as possible on Day 1 following provision of informed consent, confirmation of eligibility, completion of all baseline assessments and confirmation of food intake.

Impact of Global Public Health Emergencies (COVID-19 and Mpox)

The COVID-19 pandemic had a significant impact on the conduct of the study including a brief period where enrolment was suspended. Public health measures, redeployment of medical staff/resources, as well as prioritisation of diagnostic testing for COVID-19 and shortage of certain medical supplies resulted in a recruitment pause in the US and led to delays in site setup, activation and initial recruitment in other countries, as well as significantly extended study timelines.

Considerations regarding the safety of participants and staff and key aspects related to data quality and laboratory procedures impacted conduct of the study. Critically, in addition to the operational aspects of the pandemic, the impact of rapidly changing local travel restrictions and quarantine measures for individual participants increased the likelihood for missed or out-of-window TOC assessments. Given that this was crucial for assessment of the primary efficacy endpoint, prior assumptions used in the initial sample size estimation were re-evaluated.

To mitigate risks associated with COVID-19, a protocol amendment was submitted, and sites expedited recruitment in-line accordingly and as local guidelines permitted. Although mitigating actions varied, typical elements included COVID-19 pre-screening measures prior to clinic visits (recent exposure, respiratory symptoms, or testing at entry) and measures to limit the risk associated with interaction (use of distancing, phone interactions, and protective equipment).

Specific changes to the protocol and planned analysis as a result of COVID-19 included the following:

- Clarification on handling out-of-window visits and unscheduled visits:
  Previously, all visits that were out-of-window were considered missed, including missed TOC visits, and no microbiological data were collected. The risk of visits happening out of the allowed time window increased due to COVID-19 and the protocol was therefore adapted to collect data outside of this window for

consideration as possible supporting data. The CSP now stipulated the collection of microbiological samples at TOC visit, even when this occurred out-of-window. In the primary analysis, participants who missed, or had an out-of-window TOC visit would still be treated as failures. A sensitivity analysis was to be carried out including the microbiological outcome for these participants. However, the majority of participants were enrolled prior to implementation of this protocol amendment thus limiting the amount of data expected to be collected from out-of-window visits.

- Deletion of *C trachomatis* assessment at Day 30 visit (exploratory endpoint) and of all *M genitalium* assessments:
  Because of COVID-19-related challenges regarding the operation of the NAAT platforms (e.g. shortages of swabs and specimen collection kits, and testing facilities requisitioned for SARS-CoV-2 testing), it was decided to delete assessments that could be removed without affecting the primary and secondary objectives. The *C trachomatis* assessment at baseline at the 3 anatomical sites was not modified due to its importance in diagnosing and treating possible co-infection. However, the *C trachomatis* assessment at Day 30 for all participants who had a positive *C trachomatis* assessment at the corresponding anatomical site at baseline was deleted in line with clinical practice. Moreover, all exploratory *M genitalium* assessments and corresponding analyses were removed.
- NAAT testing allowed with other platforms:
  The initial NAAT testing platform to be used at all laboratories in the study was from Hologic Inc (Marlborough, Massachusetts, US). As components of the NAAT testing platform (e.g. polymerase chain reaction reagents) were common to SARS-CoV-2 testing, chronic shortages occurred. In mitigation, the possibility of using a different, FDA-approved NAAT platform was added to the CSP and a list of acceptable NAAT platforms was added to the laboratory manual.
- Other COVID-19-related amendments:
  Firstly, the possibility of having remote site monitoring visits was added to ensure continuity of quality oversight and monitoring due to travel bans or limited access to sites. Secondly, as aerosol/airborne transmission of COVID-19 is the main transmission mechanism, the option for participants to self-collect pharyngeal specimens under supervision of trained staff was added to the CSP.

A multi-country outbreak of Mpox started in early 2022 and was declared a public health emergency by WHO on 23 July 2022. Although several STI clinics in the USA and EU involved in the study were directly affected through short-term redeployment of resources as vaccination centres, etc, the overall effect of the Mpox outbreak on the conduct of the study was unquantified but thought to be limited. No protocol deviations were reported as attributable to Mpox and the overall impact on recruitment rate was unknown. No Mpox-related changes were made to the CSP.

Changes to the Planned Analysis

The initial study design used an assumption that the microbiological cure rate at TOC for urogenital gonorrhoea would be similar between the comparator arm (combination therapy - single IM 500 mg dose of ceftriaxone + single 1 g oral dose of azithromycin) and the interventional arm (single oral 3 g dose of zoliflodacin). The initial NI margin was set at 10% following the US FDA Guidance[1] but was changed to 12% following re-evaluation of the assumptions used in the sample size calculation, a review of a recent meta-analysis of *N gonorrhoeae* clinical trial data (Mitrani-Gold 2022), and consultation with the US FDA, after which it was agreed that a NI margin of 12% for the primary endpoint could be considered.

Therefore, the sample size calculation in the CSP was amended to propose that a single oral 3 g dose of zoliflodacin would be considered as non-inferior to a combination of a single IM 500 mg dose of ceftriaxone and a single 1 g oral dose of azithromycin if the upper bound of the 2-sided 95% CI for the urogenital microbiological cure rate of the combination therapy minus zoliflodacin was 12% or less.

Furthermore, the trial was conducted for the most part during the high peaks of the COVID-19 pandemic and therefore, monitoring of blinded, pooled data was undertaken to assess the performance of the study against assumptions used in the initial sample size determination, described in detail in the protocol, in these higher risk circumstances. When approximately 70% of participants were enrolled, the proportion of participants ineligible for the Micro-ITT population was estimated to be approximately 20% (vs 35% in initial assumptions), and the proportion of nonassessable outcomes due to missed TOC assessments at 10% (foreseen at 15% in initial assumptions). Using these estimations, a blinded, pooled data analysis was performed, considering the 4% treatment difference and a 12% non-inferiority margin. It was concluded that 696 participants evaluable for the Micro-ITT analysis set (464 in the intervention arm and 232 in the control arm, with a two-sided α of 5% and 2:1 allocation ratio) would provide approximately 90% power to show that a single oral 3 g dose of zoliflodacin is non-inferior to a combination of a single IM 500 mg dose of ceftriaxone and a single 1 g oral dose of azithromycin with respect to urogenital microbiological cure rate at TOC at the urogenital anatomical site. The

number of participants to be randomised of up to approximately 928 participants remained unchanged, as did the number of participants to be enrolled, estimated to be around 1092.

Schedule of assessments

Timing (window):	Day 1 (–1 day)[a]	Day 1[a]		Day 3 (–1 day)	Day 6 (±2 days)[b]	Day 30 (±3 days)	Early withdrawal/ unscheduled visit[c]
Visit type:	Screening	Baseline	Dosing	Phone call	TOC	EOT	
**Screening assessments**							
Informed consent[d]	X						
Demographics[e]	X						
Medical history	X						
Sexual history[f]	X			X	X	X	X
Targeted physical examination[g]	X[h]				X	X	X
Pregnancy test	X				X	X	X
Assessment of eligibility	X						
**Randomisation**	X						
**Laboratory investigations**							
HIV testing[i]		X					
FBC and differentials		X			X	X	X
Liver function tests[j]		X			X	X	X
Creatinine and eGFR		X			X	X	X
Urethral/endocervical Gram stain and microscopy examination[k]		X					
**Efficacy outcomes**							
Urogenital *N gonorrhoeae* culture and NAAT		X			X[l]	X[m]	
Rectal *N gonorrhoeae* culture and NAAT		X			X[l]	X[m]	
Pharyngeal *N gonorrhoeae* culture and NAAT		X			X[l]	X[m]	
Antimicrobial susceptibility testing[n]		X			X	X	
NAAT for *C trachomatis* (urogenital)		X					
NAAT for *C trachomatis* (rectal)		X					
NAAT for *C trachomatis* (pharyngeal)		X					
**Food intake[o]**			X				
**Trial treatment administration**			X				
**Adverse events**	◄────────────────►						
**Prior/concomitant medications**	◄────────────────►						

[a]Assessments for Day 1 could be performed over 2 days if all baseline assessments and dosing were performed on the second day. Day 1 is the day of dosing. [b]In case any of the *N gonorrhoeae* microbiological test results for a specific anatomical site were not available prior to the

collection of the TOC visit specimens, both *N gonorrhoeae* culture and NAAT microbiology specimens were to be collected for that anatomical site and microbiological evaluations were to be performed. [c]If a participant had completed scheduled visits as per protocol and returned to the clinic on an additional occurrence on a day that did not correspond to a scheduled trial visit, staff were to manage them as per their routine practice. In such cases, no assessment was mandated as per the protocol. [d]Included counselling on sexual risk behaviour, and partner(s) notification and treatment. [e]Included date of birth, gender, sex at birth, ethnicity. [f]By use of the sexual history questionnaires provided. [g]Included clinical assessment of gonorrhoea. [h]Screening physical examination also functioned as baseline. [i]HIV testing was proposed, but not mandatory for inclusion in the study. An HIV test result obtained on the day of enrolment could be used as baseline assessment. Participants with a documented positive HIV diagnosis or documented prescription of ART did not need to be tested again. [j]Alanine transaminase and total and direct bilirubin (direct bilirubin was only required if total bilirubin was elevated). [k]A Gram stain result obtained on the day of enrolment could be used as a baseline assessment. [l]In cases where both baseline *N gonorrhoeae* NAAT and *N gonorrhoeae* culture results were negative, no further samples were to be taken at subsequent visits. This applied to each anatomical site, respectively. [m]Samples were only to be taken if: 1) symptoms at any anatomical site were present. If symptoms at any anatomical site were present, samples were to be taken from all three anatomical sites for *N gonorrhoeae* culture and *N gonorrhoeae*/*C trachomatis* NAAT; 2) a negative culture and a positive *N gonorrhoeae* NAAT result was obtained at TOC. This applied to each anatomical site, respectively. [n]Only for culture samples with confirmed identification of *N gonorrhoeae*. [o]Food was to be taken before trial treatment: participants who had not eaten a substantial meal before their Day 1 assessments were provided with a moderate calorie, moderate fat meal, to be consumed within 1 hour (if >75 kg body weight) or 2 hours (if ≤75 kg body weight) prior to dosing.

ART=anti-retroviral therapy; eGFR=estimated glomerular filtration rate; EOT=end of trial; FBC=full blood count; HIV=human immunodeficiency virus; NAAT=nucleic acid amplification test; TOC=test of cure.

Inclusion and exclusion criteria

Participants provided written informed consent. Participants unable to read required a relative or impartial witness to be present during informed consent discussions, and could consent orally or if capable, by signing (or thumbprint) and dating the information and consent form. Adolescent participants (age ≥12 and <18 years) were consented according to local practice in their country of origin. If required by local ethics guidance, consent was sought from both the participant and a legal representative. In countries where the status of emancipated minors is recognised, consent was sought from the participant directly.

Inclusion criteria

Participants were required to meet all the following criteria to be eligible for enrolment into the trial:

1. ≥12 years old (if enrolment of minors agrees with local regulations and ethics guidance).
2. ≥35 kilograms.
3. Signs and symptoms consistent with urethral or endocervical gonorrhoea OR,

   Urethral or endocervical uncomplicated gonorrhoea as determined by either a positive culture or nucleic acid amplification test (NAAT) or Gram stain or methylene blue/gentian violet stain in the past 14 days prior to screening OR,

   Unprotected sexual contact with an individual confirmed to be infected with *Neisseria gonorrhoeae* in the past 14 days prior to screening (positive NAAT, Gram stain, methylene blue/gentian violet stain or culture).
4. For females of child-bearing potential, a negative urine pregnancy test at screening.
5. For females of child-bearing potential, use of a highly effective method of contraception at the time of investigative medicinal product (administration on Day 1: contraception methods which may be considered as highly effective) and until at least 28 days after treatment. Females on oral contraceptives must also use a barrier contraception method during participation in the trial.
6. For males with a female partner of child-bearing age, willingness to delay conception during the trial and for 28 days after treatment.
7. Willingness to comply with the trial protocol.
8. For participants in the pharmacokinetic (PK) sub-study: willingness to undergo HIV testing. *(Note: A pharmacokinetic sub-study was conducted and will be reported elsewhere)*
9. Willingness to abstain from sexual intercourse or use condoms for vaginal, anal, or oral sex from enrolment until the end of trial (end of trial [EOT]) visit.
10. Willingness and ability to give written informed consent, or be consented by a legal representative, or provide assent and parental consent (for minors, as appropriate).

Exclusion criteria

The presence of any of the following excluded a potential participant from trial enrolment:

1. Confirmed or suspected complicated or disseminated gonorrhoea.
2. Pregnant or breastfeeding women.
3. Known concomitant infection which would require immediate additional systemic antibiotics with activity against *N gonorrhoeae*.
4. Use of any systemic or intravaginal antibiotics with activity against *N gonorrhoeae* within 30 days prior to screening.
5. Use of systemic corticoid drugs or other immunosuppressive therapy within 30 days prior to screening.
6. Use of moderate or strong CYP3A4 inducers (e.g., efavirenz, rifampicin, carbamazepine, phenobarbital) within 30 days or five half-lives of the drug, whichever is greater, prior to screening.
7. Cytotoxic or radiation therapy within 30 days prior to screening.
8. Known chronic renal, hepatic, hematologic impairment or other condition interfering with the absorption, distribution, or elimination of the drug based on medical history and physical examination.

9. History of urogenital sex-reassignment surgery.
10. Immunosuppression as evidenced by medical history, clinical examination, or a recent (≤1 month) CD4 count <200 cells/µL.
11. Known clinically relevant cardiac pro-arrhythmic conditions such as cardiac arrhythmia, congenital or documented QT prolongation.
12. Known history of severe allergy to cephalosporin, penicillin, monobactams, carbapenems or macrolide antibiotics.
13. Known or suspected allergies or hypersensitivities to lidocaine, methylparaben, lactose, or any of the components of the study drugs.
14. Receipt or planned receipt of an investigational product in a clinical study within 30 days or five half-lives of the drug, whichever is greater, prior to screening until end of participation in this clinical trial.
15. History of alcohol or drug abuse within 12 months prior to screening which would compromise trial participation in the judgement of the investigator.
16. Severe medical or psychiatric condition which, in the opinion of the investigator, may increase the risk associated with trial participation or may interfere with the interpretation of trial results or affect the individual's ability to provide informed consent.
17. Individuals whom, in the judgement of the investigator, are unlikely or unable to comply with this trial protocol.
18. Previous randomisation in this clinical trial.
19. Use of moderate or strong CYP3A4 inhibitors within 30 days or five half-lives of the drug, whichever is greater, prior to screening.

Microbiological procedures and assessments

Brief details on microbiological procedures and evaluations are provided in the paper; additional information is provided below. The definition of microbiological outcomes used by the local and central laboratories are presented in the table that follows.

- Specimens were to be collected by trained study staff, except pharyngeal specimens which could be self-collected following a protocol amendment in response to the COVID-19 pandemic. First, a swab for *N gonorrhoeae* culture was to be taken followed by bedside inoculation of *N gonorrhoeae* selective agar plates and incubation at 35–37°C in a humidified 5–10% CO_2-enriched atmosphere until transport to the local laboratory (during which CO_2 conditions were maintained).
- Suspect colonies were subject to two of the three identification tests namely Gram's stain, oxidase, and superoxol tests. Presumed *N gonorrhoeae* positive cultures were processed, and bacterial isolates frozen in duplicate at the local laboratories.
- Confirmatory identification of *N gonorrhoeae* using Maldi-TOF MS/MS (Bruker, Germany) by the central laboratory constituted definitive data for the primary outcome of the trial.
- The MIC, defined as the lowest concentration of an antimicrobial that inhibited the visible growth of a microorganism after overnight incubation, was determined at the central laboratory for the following antibiotics: zoliflodacin, azithromycin, cefixime, ceftriaxone, ciprofloxacin, gentamicin, tetracycline, and spectinomycin.
- Presence of nucleic acid was detected with NAAT using swabs taken at the same anatomical sites after the swab for culture. Commercially available kits were used to assess the presence of *N gonorrhoeae*, *C trachomatis*, and/or *M genitalium* (when performed) from the same clinical specimen. All laboratories used an NAAT platform that had the ability to diagnose a *N gonorrhoeae* and *C trachomatis* infection in the same assay using one swab and a single test run, and was FDA approved. Examples of NAATs fulfilling these requirements include Aptima Combo 2® assay for CT/NG on Tigris or Panther platforms from Hologic, Cobas® CT/NG assay from Roche Diagnostics, RealTime CT/NG assay from Abbott and Xpert® CT/NG from Cepheid.
- Whole genome sequencing (WGS) was conducted after end of trial, as an exploratory objective, on all *N gonorrhoeae* isolates cultured at the TOC and EOT visits, as well as on corresponding pre-treatment baseline isolates from the respective participants and on baseline isolates with zoliflodacin MIC >0·25 µg/mL. The WGS analyses will be reported separately in detail (manuscript in preparation).

Safety assessments

- Safety and tolerability were assessed through direct enquiry at scheduled visits as well as spontaneous reporting of adverse events up to Day 30.
- Treatment-emergent adverse event (TEAEs), as determined by the investigators, were assessed for causality and severity, and reported by the investigators using electronic case report forms.
- Safety blood samples were collected on Day 6 and Day 30 per schedule of assessment.
- A data and safety monitoring board comprising four members who were independent of the investigators and the sponsor was constituted before participant enrolment was initiated.
- All participants were contacted on Day 3 (–1 day) by phone for safety assessment and to collect sexual behaviour data since time of dosing.
- On Day 6 (±2) (test of cure [TOC]), participants attended clinic for repeat safety assessments, collection of sexual behaviour data, and microbiological swabs for culture/AST and NAAT, where indicated per the sampling schedule.
- A final follow-up visit was conducted on Day 30 (±3) (EOT) where repeat safety assessments, collection of sexual behaviour data and microbiological swabs for culture/AST and NAAT, where indicated per the sampling schedule (pp 9-10) and presence of symptoms (p 15).

Use of microbiological results for subsequent clinical management

Microbiology test results obtained from the local laboratory for individual participants could dictate their ongoing clinical management, including the subsequent schedule for obtaining further samples. Options, depending on test outcomes, are shown below.

<table>
<tr><th rowspan="2">Timepoint</th><th rowspan="2">Assessment</th><th colspan="2">Action if result is:</th></tr>
<tr><th>Positive</th><th>Negative</th></tr>
<tr><td rowspan="2">Baseline results[a,b]</td><td>N gonorrhoeae culture and NAAT</td><td>If either culture and/or NAAT is/are positive, take further swabs for both culture and NAAT at TOC</td><td>If both N gonorrhoeae culture and NAAT are negative, no further swabs to be taken for N gonorrhoeae in the trial</td></tr>
<tr><td>C trachomatis NAAT</td><td>• Treat as per standard of care at TOC after collection of all specimens if participant was randomised to zoliflodacin arm
• No further swabs to be taken for C trachomatis (except if participant is symptomatic at Day 30)</td><td>No further swabs to be taken for C trachomatis (except if participant is symptomatic at Day 30)</td></tr>
<tr><td rowspan="2">TOC results</td><td rowspan="2">N gonorrhoeae culture and NAAT</td><td>• If N gonorrhoeae culture was positive, treat as per standard of care
• No further swabs to be taken for N gonorrhoeae[c]</td><td>If both N gonorrhoeae culture and NAAT are negative, no further swabs to be taken for N gonorrhoeae in the trial (except if participant is symptomatic at Day 30)[a,d]</td></tr>
<tr><td colspan="2">If N gonorrhoeae culture is negative and N gonorrhoeae NAAT is positive at TOC, both N gonorrhoeae culture and N gonorrhoeae NAAT to be done at Day 30[a]</td></tr>
<tr><td>Day 30 results[d]</td><td>N gonorrhoeae culture and N gonorrhoeae/ C trachomatis NAAT</td><td>Treat as per standard of care if any of the test results is positive</td><td>NA</td></tr>
</table>

[a]Applies to each of the three anatomical sites independently.
[b]In the event that baseline results are not available at the time of the TOC visit, all swabs from each of the three anatomical sites should be taken.
[c]If *N gonorrhoeae* culture is positive in any of the three anatomical sites, no further swabs need be taken from any site at Day 30, except if participant is symptomatic at Day 30.
[d]If a participant presents with signs and/or symptoms of an *N gonorrhoeae* infection at Day 30, swabs for *N gonorrhoeae* culture and *N gonorrhoeae/ C trachomatis* NAAT should be collected from all three anatomical sites.

NA=not applicable; NAAT=nucleic acid amplification test; TOC=test of cure.

Objectives and endpoints

Objectives	Endpoints
**Primary**	
To assess the efficacy of a single, oral, 3 g dose of zoliflodacin compared to a combination of a single IM 500 mg dose of ceftriaxone and single, oral, 1 g dose of azithromycin for the treatment of uncomplicated urogenital gonorrhoea.	Microbiological cure as determined by culture at urethral or endocervical sites at TOC (Day 6±2)
**Secondary**	
To assess the safety and tolerability of a single, oral, 3 g dose of zoliflodacin compared to a single IM 500 mg dose of ceftriaxone and single, oral, 1 g dose of azithromycin in participants with uncomplicated gonorrhoea.	Incidence, severity, causality and seriousness of treatment-emergent adverse events and the evaluation of changes from baseline in safety laboratory test results and physical examinations
To determine microbiological cure rate of pharyngeal gonorrhoea after administration of a single, oral, 3 g dose of zoliflodacin compared to a combination of a single IM 500 mg dose of ceftriaxone and a single, oral, 1 g dose of azithromycin.	Proportion of participants with microbiological cure as determined by culture at pharyngeal sites at TOC (Day 6±2)
To determine microbiological cure rate of rectal gonorrhoea after administration of a single, oral, 3 g dose of zoliflodacin compared to a combination of a single IM 500 mg dose of ceftriaxone and a single, oral, 1 g dose of azithromycin.	Proportion of participants with microbiological cure as determined by culture at rectal sites at TOC (Day 6±2)
To determine the clinical cure rate of symptomatic gonorrhoea in male participants after administration of a single, oral, 3 g dose of zoliflodacin compared to a combination of a single IM 500 mg dose of ceftriaxone and a single, oral, 1 g dose of azithromycin.	Proportion of male participants with clinical cure at TOC (Day 6±2)
To determine the microbiological cure rate of urogenital gonorrhoea among female and male participants, after administration of a single, oral, 3 g dose of zoliflodacin compared to a combination of a single IM 500 mg dose of ceftriaxone and a single, oral, 1 g dose of azithromycin.	Proportion of female and male participants with microbiological cure as determined by culture at endocervical or urethral site at TOC (Day 6±2)
To determine the microbiological cure rate of *N gonorrhoeae* at urethral or endocervical sites in the subset of participants with pre-existing resistance to antibiotics commonly used for *N gonorrhoeae* treatment (including to ceftriaxone alone, to azithromycin alone and to both).	Proportion of participants with microbiological cure as determined by culture at urethral or endocervical sites at the TOC visit and for whom the baseline AST profile indicated pre-existing resistance to antibiotics commonly used for *N gonorrhoeae* treatment (including to ceftriaxone, to azithromycin alone and to both)
To determine the AST profile of gonococcal strains isolated from participants with uncomplicated gonorrhoea at baseline and the TOC visit.	AST profile of gonococcal strains isolated at baseline and at TOC (Day 6±2)
To determine the eradication rate of *N gonorrhoeae* nucleic acid from urethral, endocervical, rectal, pharyngeal specimens after administration of a single, oral, 3 g dose of zoliflodacin compared to a combination of a single IM 500 mg dose of ceftriaxone and a single, oral, 1 g dose of azithromycin.	• Proportion of participants with a negative *N gonorrhoeae* NAAT from urethral or endocervical sites at TOC (Day 6±2)<br>• Proportion of participants with a negative *N gonorrhoeae* NAAT from oropharyngeal sites at TOC (Day 6±2)<br>• Proportion of participants with a negative *N gonorrhoeae* NAAT from rectal sites at TOC (Day 6±2)

AST=antimicrobial susceptibility test; EOT=end of trial; HIV=human immunodeficiency virus; IM=intramuscular; NAAT=nucleic acid amplification test; TOC=test of cure.

Analysis sets

Randomised population: All participants who were randomised.

Micro-ITT population: The micro-ITT population for urogenital, rectal, or pharyngeal populations included all randomised participants who had a positive *N gonorrhoeae* culture from the relevant anatomical site at baseline and whose baseline AST result showed no pre-existing resistance to both ceftriaxone and azithromycin (i.e., participants with ceftriaxone MIC >0·25 µg/mL and azithromycin MIC >1 µg/mL were excluded from the population). It included participants who did not comply with trial treatment, those who were lost to follow-up, those who were nonassessable for microbiological outcome, or who received antibacterial therapy with activity against *N gonorrhoeae* prior to the TOC visit. A participant's eligibility for the micro-ITT population depended on the anatomical site in question. Susceptibility to ceftriaxone and azithromycin was defined as per CLSI standards.[2] The micro-ITT population was derived by anatomical site at baseline. For example, participants who received the trial treatment and were found to have a negative *N gonorrhoeae* culture at the urethral or endocervical site at baseline visit were excluded from the micro-ITT population and thus from the primary efficacy endpoint analysis and secondary analyses corresponding to the urethral or endocervical sites. Participants who received trial product and were found to be *N gonorrhoeae* culture negative at the rectal or pharyngeal site at baseline visit were excluded from the micro-ITT population for the corresponding secondary efficacy endpoint analyses at the rectal or pharyngeal sites. The micro-ITT population was used for the evaluation of the efficacy assessments.

Modified micro-ITT population: The modified micro-ITT population (defined separately for urogenital, rectal, or pharyngeal body site) included all randomised participants who had a positive *N gonorrhoeae* culture from the relevant anatomical site at baseline (regardless of pre-existing resistance to both ceftriaxone and azithromycin).

Clinical cure population: For the efficacy analyses of clinical cure rate, the clinical cure population included all participants assigned male at birth included in the micro-ITT (urogenital) population and who had at least one sign or symptom of urethral gonorrhoea at baseline.

Safety population: The safety population included all randomised participants who received any part of trial treatment.

Evaluable population: The evaluable population (defined separately for urogenital, rectal, or pharyngeal body site) included all randomised participants who had a positive *N gonorrhoeae* culture at baseline and whose baseline AST result showed no pre-existing resistance to both ceftriaxone and azithromycin, who did not vomit within 30 minutes of administration of zoliflodacin or azithromycin and who had a *N gonorrhoeae* culture result at the TOC visit.

Per-Protocol (PP) population: The PP efficacy population (defined separately for urogenital, rectal, or pharyngeal body site) included all participants in the micro-ITT population who met all inclusion/exclusion criteria, complied with trial treatment, did not vomit within 30 minutes of administration of zoliflodacin or azithromycin, did not receive any systemic antibiotic with known activity against *N gonorrhoeae* prior to TOC visit, did not receive any of the prohibited medications, abstained from sexual intercourse or used condoms for vaginal, anal and oral sex prior to TOC, and returned to the trial site for the TOC visit within the specified window (Day 6±2). Manual data review to determine evaluability and inclusion within the PP analysis sets was performed on blinded data by the Evaluability Review Committee (ERC), which included statistical, medical, and microbiological representation. The ERC was responsible for reviewing the data as described in the Evaluability Review Plan.

Clinical-PP population: The Clinical-PP population included participants assigned male at birth in the PP population with at least one sign or symptom of gonorrhoea at baseline and with an evaluable clinical outcome.

Definitions used for microbiological assessments and nucleic acid amplification tests

**Laboratory location**<br>Analysis/result	**Definition**
**Local laboratory (presumptive results)**	
Culture/presumptive positive for *N gonorrhoeae*	Colony morphologies suggestive of gonococci (generally small, translucent, raised, grey, and mucoid with entire margins) that show Gram negative diplococci and are positive with oxidase and/or superoxol tests
Culture/negative for *N gonorrhoeae*	Culture plates that do not show colonies suggestive of gonococci at the end of 72 hours incubation
Culture/indeterminate for *N gonorrhoeae*	Culture plates overgrown with fungi, yeast, *Proteus,* or other contaminating organisms that prevent isolation of pure *N gonorrhoeae*, leading to the inability to determine presence or absence of *N gonorrhoeae*
**Central laboratory confirmation**	
Culture/positive for *N gonorrhoeae*	Colony morphologies consistent with *N gonorrhoeae* confirmed to genus and species level by an automated identification system (MALDI-TOF MS, Bruker, Germany) as *N gonorrhoeae*
Culture/not *N gonorrhoeae*	Colony morphologies consistent with *N gonorrhoeae* confirmed to genus and species level by an automated identification system (MALDI-TOF MS, Bruker, Germany) as another species of Neisseria or a non-Neisseria genus
Culture/no growth	Culture showed no growth of bacteria
**Local laboratory**	
NAAT/positive or negative for *C trachomatis/N gonorrhoeae*	Results automatically interpreted by the software of the NAAT used and presented as individual *C trachomatis* and *N gonorrhoeae*
NAAT/indeterminate for *C trachomatis/N gonorrhoeae*	Results automatically interpreted by the software of the NAAT used and presented as individual *C trachomatis* and *N gonorrhoeae*, reported as indeterminate after confirming in a repeat analysis

MALDI-TOF MS= Matrix-assisted laser desorption/ionization time-of-flight mass spectrometry; NAAT=nucleic acid amplification test.

Supplementary Results

Table S1. Analysis datasets summary

Analysis set<br>Excluded<br>Reason	Zoliflodacin (N=621) n (%)	Ceftriaxone + azithromycin (N=309) n (%)	Overall (N=1011) n (%)
**All randomised**	**621 (100)**	**309 (100)**	**930 (92·0)**
Excluded (screening failures)	NA	NA	81 (8·0)
Not randomised	NA	NA	81 (100)
**Micro-ITT (urogenital)[a]**	**506 (81·5)**	**238 (77·0)**	**744 (80·0)**
Excluded[b]	115 (18·5)[c]	71 (23·0)[c]	186 (20·0)[c]
No positive *N gonorrhoeae* culture at urogenital site at baseline	115 (100)[c]	71 (100)[c]	186 (100)[c]
Pre-existing resistance to ceftriaxone + azithromycin at baseline	0	0	0
**Micro-ITT (pharyngeal)[a]**	**53 (8·5)**	**28 (9·1)**	**81 (8·7)**
Excluded[b]	568 (91·5)	281 (90·9)	849 (91·3)
No positive *N gonorrhoeae* culture at pharyngeal site at baseline	568 (100)	281 (100)	849 (100)
Pre-existing resistance to ceftriaxone + azithromycin at baseline	0	0	0
**Micro-ITT (rectal)[a]**	**79 (12·7)**	**35 (11·3)**	**114 (12·3)**
Excluded[b]	542 (87·3)	274 (88·7)	816 (87·7)
No positive *N gonorrhoeae* culture at rectal site at baseline	542 (100)	274 (100)	816 (100)
Pre-existing resistance to ceftriaxone + azithromycin at baseline	0	0	0
**Clinical cure**	**460 (74·1)**	**220 (71·2)**	**680 (73·1)**
Excluded[b]	161 (25·9)	89 (28·8)	250 (26·9)
Subject not in micro-ITT set for any site	76 (47·2)	45 (50·6)	121 (48·4)
Subject not assigned male at birth	77 (47·8)	38 (42·7)	115 (46·0)
No signs or symptoms of urethral gonorrhea at baseline	22 (13·7)	13 (14·6)	35 (14·0)
**Safety**	**619 (99·7)**	**308 (99·7)**	**927 (99·7)**
Excluded[b]	2 (0·3)	1 (0·3)	3 (0·3)
Subject not dosed	2 (100)	1 (100)	3 (100)
**Evaluable (urogenital)**	**475 (76·5)**	**229 (74·1)**	**704 (75·7)**
Excluded[b]	146 (23·5)	80 (25·9)	226 (24·3)
Not in micro-ITT set for urogenital site	115 (78·8)	71 (88·8)	186 (82·3)
Vomited within 30 minutes of oral administration	0	0	0
No *N gonorrhoeae* culture for anatomical site at TOC (incl window)	31 (21·2)	9 (11·3)	40 (17·7)
**Evaluable (pharyngeal)**	**46 (7·4)**	**23 (7·4)**	**69 (7·4)**
Excluded[b]	575 (92·6)	286 (92·6)	861 (92·6)
Not in micro-ITT set for pharyngeal site	568 (98·8)	281 (98·3)	849 (98·6)
Vomited within 30 minutes of oral administration	0	0	0
No *N gonorrhoeae* culture for anatomical site at TOC (incl window)	7 (1·2)	5 (1·7)	12 (1·4)
**Evaluable (rectal)**	**72 (11·6)**	**31 (10·0)**	**103 (11·1)**
Excluded[b]	549 (88·4)	278 (90·0)	827 (88·9)
Not in micro-ITT set for rectal site	542 (98·7)	274 (98·6)	816 (98·7)
Vomited within 30 minutes of oral drug administration	0	0	0

**Analysis set**<br>Excluded<br>Reason	**Zoliflodacin (N=621) n (%)**	**Ceftriaxone + azithromycin (N=309) n (%)**	**Overall (N=1011) n (%)**
No *N gonorrhoeae* culture for anatomical site at TOC (incl window)	7 (1·3)	4 (1·4)	11 (1·3)
**Per-protocol (urogenital)**	**452 (72·8)**	**219 (70·9)**	**671 (72·2)**
Excluded[b]	169 (27·2)	90 (29·1)	259 (27·8)
Not in micro-ITT site for urogenital site	115 (68·0)	71 (78·9)	186 (71·8)
Did not meet all inclusion/exclusion criteria	3 (1·8)	1 (1·1)	4 (1·5)
Did not comply with trial treatment	0	1 (1·1)	1 (0·4)
Did not abstain from sexual intercourse or use condoms prior to TOC	11 (6·5)	4 (4·4)	15 (5·8)
Vomited within 30 minutes of oral drug administration	0	0	0
Received a systemic antibiotic with known activity against NG prior to TOC	7 (4·1)	4 (4·4)	11 (4·2)
Received a prohibited medication	12 (7·1)	4 (4·4)	16 (6·2)
Did not attend TOC visit within specified window	27 (16·0)	8 (8·9)	35 (13·5)
Other key PD that would affect efficacy outcome	4 (2·4)	2 (2·2)	6 (2·3)
**Per-protocol (pharyngeal)**	**46 (7·4)**	**23 (7·4)**	**69 (7·4)**
Excluded[b]	575 (92·6)	286 (92·6)	861 (92·6)
Not in micro-ITT site for pharyngeal site	568 (98·8)	281 (98·3)	849 (98·6)
Did not meet all inclusion/exclusion criteria	0	0	0
Did not comply with trial treatment	0	0	0
Did not abstain from sexual intercourse or use condoms prior to TOC	3 (0·5)	0	3 (0·3)
Vomited within 30 minutes of oral drug administration	0	0	0
Received a systemic antibiotic with known activity against *N gonorrhoeae* prior to TOC	1 (0·2)	1 (0·3)	2 (0·2)
Received a prohibited medication	2 (0·3)	1 (0·3)	3 (0·3)
Did not attend TOC visit within specified window	4 (0·7)	3 (1·0)	7 (0·8)
Other key PD that would affect efficacy outcome	0	1 (0·3)	1 (0·1)
**Per-protocol (rectal)**	**64 (10·3)**	**31 (10·0)**	**95 (10·2)**
Excluded[b]	557 (89·7)	278 (90·0)	835 (89·8)
Not in micro-ITT site for rectal site	542 (97·3)	274 (98·6)	816 (97·7)
Did not meet all inclusion/exclusion criteria	2 (0·4)	0	2 (0·2)
Did not comply with trial treatment	0	0	0
Did not abstain from sexual intercourse or use condoms prior to TOC	7 (1·3)	0	7 (0·8)
Vomited within 30 minutes of oral drug administration	0	0	0
Received a systemic antibiotic with known activity against *N gonorrhoeae* prior to TOC	1 (0·2)	1 (0·4)	2 (0·2)
Received a prohibited medication	2 (0·4)	1 (0·4)	3 (0·4)
Did not attend TOC visit within specified window	6 (1·1)	3 (1·1)	9 (1·1)
**Clinical Per-protocol**	**410 (66·0)**	**201 (65·0)**	**611 (65·7)**
Excluded[b]	211 (34·0)	108 (35·0)	319 (34·3)
Subject not in per-protocol set for any site	125 (59·2)	61 (56·5)	186 (58·3)
Not assigned male at birth	77 (36·5)	38 (35·2)	115 (36·1)

**Analysis set**<br>Excluded<br>Reason	**Zoliflodacin (N=621) n (%)**	**Ceftriaxone + azithromycin (N=309) n (%)**	**Overall (N=1011) n (%)**
No sign or symptom of urethral gonorrhea at baseline	6 (2·8)	6 (5·6)	12 (3·8)
Subject did not have evaluable clinical outcome	9 (4·3)	9 (8·3)	18 (5·6)

[a]The micro-ITT population and modified micro-ITT population (not shown) were identical. [b]Percentages in subcategories for exclusion are calculated using the total number excluded as the denominator. [c]Three participants that had been randomised, but not treated (2 in the zoliflodacin arm, 1 in the ceftriaxone+azithromycin arm as displayed in Figure 1) are mentioned in this table as part of the participants excluded from micro-ITT as they did not have samples taken, so no positive *N gonorrhoeae* urogenital culture at baseline.

ITT=intention-to-treat; micro=microbiological; PD=protocol deviation; TOC=test of cure.

Baseline characteristics

Table S2. **Additional participant demographics and baseline characteristics: randomised population**

	**Zoliflodacin (n=621)**	**Ceftriaxone + azithromycin (n=309)**
**Age group, n (%)**		
<18 years	12 (1.9)	2 (0.6)
18 to 64 years	606 (97.6)	306 (99.4)
≥65 years	3 (0.5)	1 (0.3)
**Body mass index (kg/m^2), mean (SD)**	23.68 (4.714)	23.26 (4.567)
**History of sexually transmitted infection(s), n (%)**		
Yes	343 (55·2)	179 (57·9)
No	278 (44·8)	130 (42·1)
**Baseline antibiotic resistance[a], n (%)**		
Resistant to ceftriaxone alone	0	0
Resistant to azithromycin alone	29 (4·7)	12 (3·9)
Resistant to both ceftriaxone and azithromycin	0	0
Susceptible to both ceftriaxone and azithromycin	476 (76·7)	225 (72·8)
**Sex worker, n (%)**		
Never	580 (93·4)	290 (93·9)
Every now and then (sometimes)	23 (3·7)	16 (5·2)
Often	17 (2·7)	2 (0·6)
Missing	1 (0·2)	1 (0·3)
**Taking PrEP medication at baseline, n (%)**		
Yes	84 (13·5)	42 (13·6)
No	537 (86·5)	267 (86·4)
**MSM, n (%)**		
Yes	198 (31·9)	105 (34·0)
No	423 (68·1)	204 (66·0)

[a]According to Clinical & Laboratory Standards Institute criteria.

MSM=men who have sex with men (i.e., assigned male at birth and usually engage in sexual contact with men or transgender women); NAAT=nucleic acid amplification test; PrEP=pre-exposure prophylaxis; SD = standard deviation.

Prior and concomitant treatments

In the safety population, prior use of medications at baseline (within 30 days prior to screening) was balanced between treatment arms. Concomitant medication use was generally balanced between treatment groups, except for concomitant use of antibacterials (43% for zoliflodacin *vs* 10% for ceftriaxone plus azithromycin); concomitant antibacterial use in the zoliflodacin group was largely driven by the use of azithromycin, ceftriaxone, and doxycycline, which were permitted at or after the TOC visit per protocol and according to the respective standard of care at each study site, for the treatment of co-infections (e.g., *C trachomatis* or *M genitalium*), as well as in cases of microbiological and/or clinical failure, and/or potential *N gonorrhoeae* re-infection.

Baseline microbiological assessments

Table S3. **Summary of microbiological evaluations by local laboratories: randomised population**

**Status**<br>**Result**	**Zoliflodacin (N=621) n (%)**	**Ceftriaxone + azithromycin (N=309) n (%)**	**Overall (N=930) n (%)**	**Zoliflodacin (N=621) n (%)**	**Ceftriaxone + azithromycin (N=309) n (%)**	**Overall (N=930) n (%)**	**Zoliflodacin (N=621) n (%)**	**Ceftriaxone + azithromycin (N=309) n (%)**	**Overall (N=930) n (%)**	**Zoliflodacin (N=621) n (%)**	**Ceftriaxone + azithromycin (N=309) n (%)**	**Overall (N=930) n (%)**
**Parameter**	***N gonorrhoeae* culture/Urogenital**			***N gonorrhoeae* NAAT/Urogenital**			***C trachomatis* NAAT/Urogenital**			***M genitalium* NAAT/Urogenital**		
Completed[a]	619	308	927	616	307	923	616	307	923	289	141	430
Negative	106 (17·1)	64 (20·8)	170 (18·3)	97 (15·7)	50 (16·3)	147 (15·9)	456 (74·0)	227 (73·9)	683 (74·0)	262 (90·7)	124 (87·9)	386 (89·8)
Positive	513 (82·9)[b]	244 (79·2)[b]	757 (81·7)[b]	516 (83·8)	255 (83·1)	771 (83·5)	157 (25·5)	78 (25·4)	235 (25·5)	27 (9·3)	17 (12·1)	44 (10·2)
Indeterminate	0	0	0	3 (0·5)	2 (0·7)	5 (0·5)	3 (0·5)	2 (0·7)	5 (0·5)	0	0	0
Not Completed[c]	2	1	3	5	2	7	5	2	7	332	168	500
	***N gonorrhoeae* culture/Rectal**			***N gonorrhoeae* NAAT/Rectal**			***C trachomatis* NAAT/Rectal**					
Completed[a]	619	308	927	616	306	922	616	306	922			
Negative	539 (87·1)	271 (88·0)	810 (87·4)	302 (49·0)	144 (47·1)	446 (48·4)	548 (89·0)	268 (87·6)	816 (88·5)			
Positive	80 (12·9)[b]	37 (12·0)[b]	117 (12·6)[b]	294 (47·7)	153 (50·0)	447 (48·5)	66 (10·7)	38 (12·4)	104 (11·3)			
Indeterminate	0	0	0	20 (3·2)	9 (2·9)	29 (3·1)	2 (0·3)	0	2 (0·2)			
Not Completed[c]	2	1	3	5	3	8	5	3	8			
	***N gonorrhoeae* culture/Pharyngeal**			***N gonorrhoeae* NAAT/Pharyngeal**			***C trachomatis* NAAT/Pharyngeal**					
Completed[a]	619	308	927	617	308	925	617	308	925			
Negative	549 (88·7)	271 (88·0)	820 (88·5)	458 (74·2)	221 (71·8)	679 (73·4)	598 (96·9)	299 (97·1)	897 (97·0)			
Positive	64 (10·3)[b]	36 (11·7)[b]	100 (10·8)[b]	150 (24·3)	83 (26·9)	233 (25·2)	19 (3·1)	9 (2·9)	28 (3·0)			
Indeterminate	6 (1·0)	1 (0·3)	7 (0·8)	9 (1·5)	4 (1·3)	13 (1·4)	0	0	0			
Not Completed[c]	2	1	3	4	1	5	4	1	5			

[a]Denominator for percentages in the ‘completed’ categories. [b]Presumptive identification at the local laboratory. [c]Denominator for percentages in the 'not completed' categories.
Micro-ITT=microbiological intention-to-treat; NAAT=nucleic acid amplification test.

***Table S4.* Comparison of *N gonorrhoeae* culture data from local laboratory vs central laboratory: randomised population**

**Local Laboratory Results**				**Central Laboratory Results**			
**Category**<br>Status<br>Result	**Zoliflodacin (N=621) n (%)**	**Ceftriaxone + azithromycin (N=309) n (%)**	**Overall (N=930) n (%)**	**Category**<br>**Status**<br>**Result**	**Zoliflodacin n (%)**	**Ceftriaxone + azithromycin n (%)**	**Overall n (%)**
***N gonorrhoeae* culture – urogenital**				***N gonorrhoeae* culture – urogenital**			
Completed[a]	619	308	927	Positive at local laboratory[b]	513	244	757
Negative	106 (17·1)	64 (20·8)	170 (18·3)	Not *N gonorrhoeae*	4 (0·8)	4 (1·6)	8 (1·1)
Positive[c]	513 (82·9)	244 (79·2)	757 (81·7)	*N gonorrhoeae* confirmed[d]	506 (98·6)	238 (97·5)	744 (98·3)
Indeterminate	0	0	0	No growth	3 (0·6)	1 (0·4)	4 (0·5)
				Not done	0	1(0·4)	1 (0·1)
Not completed[e]	2	1	3				
Not done	2 (100)	1 (100)	3 (100)	NA			
Not required	0	0	0				
Sample rejected	0	0	0				
***N gonorrhoeae* culture - rectal**				***N gonorrhoeae* culture – rectal**			
Completed[a]	619	308	927	Positive at local laboratory[b]	80	37	117
Negative	539 (87·1)	271 (88·0)	810 (87·4)	Not *N gonorrhoeae*	0	2 (5·4)	2 (1·7)
Positive[c]	80 (12·9)	37 (12·0)	117 (12·6)	*N gonorrhoeae* confirmed[d]	79 (98·8)	35 (94·6)	114 (97·4)
Indeterminate	0	0	0	No growth	1 (1·3)	0	1 (0·9)
Not completed[e]	2	1	3				
Not done	2 (100)	1 (100)	3 (100)	NA			
Not required	0	0	0				
Sample rejected	0	0	0				
***N gonorrhoeae* culture - pharyngeal**				***N gonorrhoeae* culture - pharyngeal**			
Completed[a]	619	308	927	Positive at local laboratory[b]	64	36	100
Negative	549 (88·7)	271 (88·0)	820 (88·5)	Not *N gonorrhoeae*	10 (15·6)	7 (19·4)	17 (17·0)
Positive[c]	64 (10·3)	36 (11·7)	100 (10·8)	*N gonorrhoeae* confirmed[d]	53 (82·8)	28 (77·8)	81 (81·0)

Local Laboratory Results				Central Laboratory Results			
**Category**<br>Status<br>Result	**Zoliflodacin (N=621) n (%)**	**Ceftriaxone + azithromycin (N=309) n (%)**	**Overall (N=930) n (%)**	**Category**<br>**Status**<br>**Result**	**Zoliflodacin n (%)**	**Ceftriaxone + azithromycin n (%)**	**Overall n (%)**
Indeterminate	6 (1·0)	1 (0·3)	7 (0·8)	No growth	1 (1·6)	1 (2·8)	2 (2·0)
Not completed[e]	2	1	3	NA			
Not done	2 (100)	1 (100)	3 (100)				
Not required	0	0	0				
Sample rejected	0	0	0				

[a]Denominator for percentages for all subcategories of Completed. [b]Denominator for percentages for all Central Laboratory results. [c]Presumptive identification at the local laboratory. [d]Confirmatory identification at the Central Laboratory on presumptive isolates from the Local Laboratory. [e]Denominator for percentages for all subcategories of Not Completed.

NA=not applicable (as there was no isolate to be tested).

Central laboratory *N gonorrhoeae* culture data were summarised in the randomised population as a post-hoc analysis

Baseline MIC distributions

Table S5. **Summary of MIC and antimicrobial susceptibility status for *N gonorrhoeae* at baseline: micro-ITT (urogenital) population**

Treatment arm (M)	Antimicrobial	MIC_{50} (µg/mL)	MIC_{90} (µg/mL)	Range (µg/mL)	CLSI[a]		EUCAST[a]	
					MIC susceptible breakpoint	Non-susceptible	MIC susceptible breakpoint	Non-susceptible
Zoliflodacin (M=505)	Azithromycin	0·12	1	≤0·06, >8	≤1	5·7%	≤1	5·7%
	Cefixime	0·008	0·06	≤0·002, >0·5	≤0·25	0·6%	≤0·125	1·2%
	Ceftriaxone	0·004	0·015	≤0·002, 0·25	≤0·25	0%	≤0·125	0·2%
	Ciprofloxacin	2	>2	≤0·0005, >2	≤0·06	74·9%	≤0·03	74·9%
	Gentamicin	8	8	1, 16	NA	NA	NA	NA
	Spectinomycin	32	32	8, 64	≤32	2·0%	≤64	0%
	Tetracycline	>4	>4	≤0·12, >4	≤0·25	93·5%	≤0·5	71·5%
	Zoliflodacin	0·06	0·12	≤0·008, 0·5	NA	NA	NA	NA
Ceftriaxone + azithromycin (M=236)	Azithromycin	0·12	1	≤0·06, >8	≤1	5·1%	≤1	5·1%
	Cefixime	0·008	0·03	≤0·002, 0·5	≤0·25	0·4%	≤0·125	1·3%
	Ceftriaxone	0·008	0·015	≤0·002, 0·12	≤0·25	0%	≤0·125	0%
	Ciprofloxacin	2	>2	0·002, >2	≤0·06	75·4%	≤0·03	75·4%
	Gentamicin	8	8	≤0·5, 16	NA	NA	NA	NA
	Spectinomycin	32	32	8, 64	≤32	0·8%	≤64	0%
	Tetracycline	>4	>4	≤0·12, >4	≤0·25	93·6%	≤0·5	75·4%
	Zoliflodacin	0·06	0·12	≤0·008, 0·25	NA	NA	NA	NA

[a]MIC breakpoints obtained from: CLSI criteria[2] and EUCAST criteria.[3]

CLSI=Clinical and Laboratory Standards Institute; EUCAST=European Committee on Antimicrobial Susceptibility Testing; M=number of participants in a micro-ITT population relevant for the specified body site without missing data; MIC=minimum inhibitory concentration; micro-ITT=microbiological intention to treat; MIC_{50}=MIC required to inhibit growth of 50% of isolates; MIC_{90}=MIC required to inhibit growth of 90% of isolates; NA=not applicable.

***Table S6.* Summary of MIC and antimicrobial susceptibility status for *N gonorrhoeae* at baseline: micro-ITT (pharyngeal) population**

Treatment arm	Antimicrobial	MIC_{50} (µg/mL)	MIC_{90} (µg/mL)	Range (µg/mL)	CLSI[a]		EUCAST[a]	
					MIC susceptible breakpoint	Non-susceptible	MIC susceptible breakpoint	Non-susceptible
Zoliflodacin (M=53)	Azithromycin	0·25	2	≤0·06, 4	≤1	11·3%	≤1	11·3%
	Cefixime	0·008	0·06	≤0·002, >0·5	≤0·25	1·9%	≤0·125	3·8%
	Ceftriaxone	0·004	0·015	≤0·002, >0·5	≤0·25	1·9%	≤0·125	0·9%
	Ciprofloxacin	2	>2	0·004, >2	≤0·06	71·7%	≤0·03	71·7%
	Gentamicin	8	8	2, 16	NA	NA	NA	NA
	Spectinomycin	32	32	≤4, 64	≤32	5·7%	≤64	0%
	Tetracycline	>4	>4	≤0·12, >4	≤0·25	96·2%	≤0·5	79·2%
	Zoliflodacin	0·06	0·25	≤0·008, 0·5	NA	NA	NA	NA
Ceftriaxone + azithromycin (M=28)	Azithromycin	0·12	2	≤0·06, 4	≤1	10·7%	≤1	10·7%
	Cefixime	0·015	0·06	≤0·002, 0·06	≤0·25	0%	≤0·125	0%
	Ceftriaxone	0·008	0·015	≤0·002, 0·015	≤0·25	0%	≤0·125	0%
	Ciprofloxacin	2	>2	0·004, >2	≤0·06	71·4%	≤0·03	71·4%
	Gentamicin	8	16	2, 16	NA	NA	NA	NA
	Spectinomycin	32	32	16, 64	≤32	7·1%	≤64	0%
	Tetracycline	>4	>4	≤0·12, >4	≤0·25	92·9%	≤0·5	75·0%
	Zoliflodacin	0·06	0·25	≤0·008, 0·25	NA	NA	NA	NA

[a]MIC breakpoints obtained from: CLSI criteria[2] and EUCAST criteria.[3]

CLSI=Clinical and Laboratory Standards Institute; EUCAST=European Committee on Antimicrobial Susceptibility Testing; M=number of participants in a micro-ITT population relevant for the specified body site without missing data; MIC=minimum inhibitory concentration; micro-ITT=microbiological intention to treat; MIC_{50}=MIC required to inhibit growth of 50% of isolates; MIC_{90}=MIC required to inhibit growth of 90% of isolates; NA=not applicable.

Table S7. Summary of MIC and antimicrobial susceptibility status for *N gonorrhoeae* at baseline: micro-ITT (rectal) population

Treatment arm	Antimicrobial	MIC_{50} (µg/mL)	MIC_{90} (µg/mL)	Range (µg/mL)	CLSI[a]		EUCAST[a]	
					MIC susceptible breakpoint	Non-susceptible	MIC susceptible breakpoint	Non-susceptible
Zoliflodacin (M=79)	Azithromycin	0·12	1	≤0·06, 2	≤1	7·6%	≤1	7·6%
	Cefixime	0·008	0·06	≤0·002, 0·5	≤0·25	2·5%	≤0·125	2·5%
	Ceftriaxone	0·008	0·015	≤0·002, 0·25	≤0·25	0%	≤0·125	2·5%
	Ciprofloxacin	2	>2	0·001, >2	≤0·06	86·1%	≤0·03	86·1%
	Gentamicin	8	8	2, 16	NA	NA	NA	NA
	Spectinomycin	32	32	8, 32	≤32	0%	≤64	0%
	Tetracycline	>4	>4	0·5, >4	≤0·25	100%	≤0·5	73·4%
	Zoliflodacin	0·06	0·12	≤0·008, 0·25	NA	NA	NA	NA
Ceftriaxone + azithromycin (M=35)	Azithromycin	0·12	1	≤0·06, 1	≤1	0%	≤1	0%
	Cefixime	0·008	0·03	≤0·002, 0·5	≤0·25	2·9%	≤0·125	2·9%
	Ceftriaxone	0·004	0·015	≤0·002, 0·06	≤0·25	0%	≤0·125	0%
	Ciprofloxacin	2	>2	0·004, >2	≤0·06	77·1%	≤0·03	77·1%
	Gentamicin	8	8	2, 16	NA	NA	NA	NA
	Spectinomycin	32	32	8, 32	≤32	0%	≤64	0%
	Tetracycline	>4	>4	≤0·12, >4	≤0·25	97·1%	≤0·5	77·1%
	Zoliflodacin	0·06	0·12	≤0·008, 0·12	NA	NA	NA	NA

[a]MIC breakpoints obtained from: CLSI criteria[2] and EUCAST criteria.[3]

CLSI=Clinical and Laboratory Standards Institute; EUCAST=European Committee on Antimicrobial Susceptibility Testing; M=number of participants in a micro-ITT population relevant for the specified body site without missing data; MIC=minimum inhibitory concentration; micro-ITT=microbiological intention to treat; MIC_{50}=MIC required to inhibit growth of 50% of isolates; MIC_{90}=MIC required to inhibit growth of 90% of isolates; NA=not applicable.

Secondary endpoints

Figure S2. **Difference in microbiological cure rate at TOC (urogenital site including sensitivity analysis populations)**

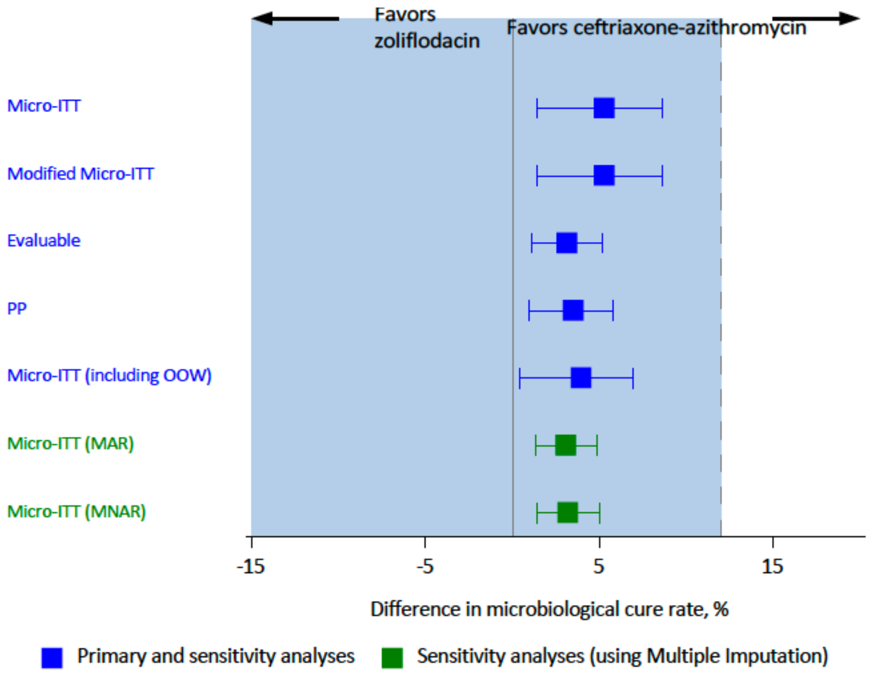

MAR=missing at random; MNAR=missing not at random; micro-ITT=microbiological intention to treat; OOW=out of window.

The dotted line at the right of the figure indicates the noninferiority margin of 12%. Data for this figure are summarised in Table 2 in the main manuscript.

Table S8. Clinical cure rate at TOC in male-at-birth participants

Analysis population (assigned male at birth)	Zoliflodacin			Ceftriaxone + azithromycin		Difference (95% CI)[c,d]
	n/N[a]	Proportion (95% CI)[b]		n/N[a]	Proportion (95% CI)[b]	
	Clinical cure rate[e]					
Clinical cure	375/460	81·5% (77·7 to 85·0)		194/220	88·2% (83·2 to 92·1)	6·66% (0·73 to 11·91)
Clinical per protocol	345/410	84·1% (80·2 to 87·5)		183/201	91·0% (86·2 to 94·6)	6·90% (1·16 to 11·91)

[a]n: number of participants with cure, N: number of participants in the analysis population and treatment arm. [b]Calculated with the Clopper Pearson method. [c]Calculated with the Newcombe score method. [d]Difference calculated as treatment difference of ceftriaxone + azithromycin minus zoliflodacin. [e]Clinical cure was defined as participants assigned male at birth experiencing resolution of signs and symptoms of urogenital gonococcal infection at TOC that were present at baseline.

CI=confidence interval; TOC=test of cure.

Table S9. **Microbiological cure rate at TOC at urogenital site in subgroups: micro-ITT and evaluable (urogenital) population**

	**Microbiological cure rate[e]**									
	**microITT population**					**Evaluable population**				
	**Zoliflodacin**		**Ceftriaxone + azithromycin**			**Zoliflodacin**		**Ceftriaxone + azithromycin**		
**Subgroup**	**n/N[a]**	**Proportion (95% CI)[b]**	**n/N[a]**	**Proportion (95% CI)[b]**	**Difference (95% CI)[c,d]**	**n/N[a]**	**Proportion (95% CI)[b]**	**n/N[a]**	**Proportion (95% CI)[b]**	**Difference (95% CI)[c,d]**
**Assigned sex at birth**										
Male	412/456	90·4% (87·3 to 92·9)	213/220	96·8% (93·6 to 98·7)	6·47% (2·45 to 9·93)	412/426	96·7% (94·5 to 98·2)	213/213	100·0% (98·3 to 100·0)	3·29% (1·08 to 5·44)
Female	48/50	96·0% (86·3 to 99·5)	16/18	88·9% (65·3 to 98·6)	–7·11% (–28·99 to 5·28)	48/49	98·0% (89·1 to 99·9)	16/16	100·0% (79·4 to 100·0%)	2·04% (-17·39 to 10·69)
**Gender**										
Male	411/455	90·3% (87·2 to 92·9)	212/219	96·8% (93·5 to 98·7)	6·47% (2·44 to 9·95)	411/425	96·7% (94·5 to 98·2)	212/212	100·0% (98·3 to 100·0)	3·29% (1·08 to 5·45)
Female	49/51	96·1% (86·5 to 99·5)	17/19	89·5% (66·9 to 98·7)	-6·60% (-27·66 to 5·40)	49/50	98·0% (89·4 to 99·9)	17/17	100·0% (80·5 to 100·0)	2·00% (-16·51 to 10·50)
**Body mass**										
≥35-≤50 kg	20/21	95·2% (76·2 to 99·9)	8/8	100·0% (63·1 to 100·0)	4·76% (-27·91 to 22·67)	20/21	95·2% (76·2 to 99·9)	8/8	100·0% (63·1 to 100·0)	4·76% (-27·91 to 22·67)
>50-≤75 kg	290/321	90·3% (86·6 to 93·3)	160/167	95·8% (91·6 to 98·3)	5·47% (0·43 to 9·76)	290/300	96·7% (94·0 to 98·4)	160/160	100·0% (97·7 to 100·0)	3·33% (0·54 to 6·03)
>75-≤100 kg	127/139	91·4% (85·4 to 95·5)	57/59	96·6% (88·3 to 99·6)	5·24% (-3·68 to 11·59)	127/131	96·9% (92·4 to 99·2)	57/57	100·0% (93·7 to 100·0)	3·05% (-3·53 to 7·59)
>100 kg	21/23	91·3% (72·0 to 98·9)	4/4	100·0% (39·8 to 100·0)	8·70% (-40·69 to 26·80)	21/21	100·0% (83·9 to 100·0)	4/4	100·0% (39·8 to 100·0)	
**HIV status**										
Positive	95/105	90·5% (83·2 to 95·3)	46/47	97·9% (88·7 to 99·9)	7·40% (-2·55 to 14·73)	95/98	96·9% (91·3 to 99·4)	46/46	100·0% (92·3 to 100·0)	3·06% (-4·91 to 8·62)
Negative	345/374	92·2% (89·1 to 94·7)	177/185	95·7% (91·7 to 98·1)	3·43% (-1·16 to 7·23)	345/356	96·9% (94·5 to 98·4)	177/177	100·0% (97·9 to 100·0)	3·09% (0·57 to 5·45)
Missing	20/27	74·1% (53·7 to 88·9)	6/6	100·0% (54·1 to 100·0)	25·93% (-15·14 to 44·68)	20/21	95·2% (76·2 to 99·9)	6/6	100·0% (54·1 to 100·0)	4·76% (-34·47 to 22·67)
**Race**										
Black or African American	241/278	86·7% (82·1 to 90·5)	125/128	97·7% (93·3 to 99·5)	10·97% (5·41 to 15·72)	241/254	94·9% (91·4 to 97·2)	125/125	100·0% (97·1 to 100·0)	5·12% (1·47 to 8·56)
White	51/57	89·5% (78·5 to 96·0)	34/38	89·5% (75·2 to 97·1)	0·00% (-14·72 to 12·36)	51/52	98·1% (89·7 to 100·0)	34/34	100·0% (89·7 o 100·0)	1·92% (-8·35 to 10·12)

	Microbiological cure rate[e]									
	microITT population					Evaluable population				
	Zoliflodacin		Ceftriaxone + azithromycin			Zoliflodacin		Ceftriaxone + azithromycin		
Subgroup	n/N[a]	Proportion (95% CI)[b]	n/N[a]	Proportion (95% CI)[b]	Difference (95% CI)[c,d]	n/N[a]	Proportion (95% CI)[b]	n/N[a]	Proportion (95% CI)[b]	Difference (95% CI)[c,d]
Asian	158/161	98·1% (94·7 to 99·6)	65/67	97·0% (89·6 to 99·6)	-1·12% (-8·49 to 2·97)	158/159	99·4% (96·5 to 100·0)	65/65	100·0% (94·5 to 100·0)	0·63% (-4·98 to 3·48)
Other	10/10	100·0% (69·2 to 100·0)	5/5	100·0% (47·8 to 100·0)		10/10	100·0% (69·2 to 100·0)	5/5	100·0% (47·8 to 100·0)	
**Region**										
Europe	42/45	93·3% (81·7 to 98·6)	15/17	88·2% (63·6 to 98·5)	-5·10% (-28·09 to 8·94)	42/43	97·7% (87·7 to 99·9)	15/15	100·0% (78·2 to 100·0)	2·33% (-18·15 to 12·06)
South Africa	188/217	86·6% (81·4 to 90·9)	111/113	98·2% (93·8 to 99·8)	11·59% (5·68 to 16·92)	188/199	94·5% (90·3 to 97·2)	111/111	100·0% (96·7 to 100·0)	5·53% (1·40 to 9·63)
Thailand	146/149	98·0% (94·2 to 99·6)	63/64	98·4% (91·6 to 100·0)	0·45% (-6·45 to 4·40)	146/147	99·3% (96·3 to 100·0)	63/63	100·0% (94·3 to 100·0)	0·68% (-5·09 to 3·75)
US	84/95	88·4% (80·2 to 94·1)	40/44	90·9% (78·3 to 97·5)	2·49% (-10·57 to 12·18)	84/86	97·7% (91·9 to 99·7)	40/40	100·0% (91·2 to 100·0)	2·33% (-6·60 to 8·09)
**Fasted status**										
Fasted	53/58	91·4% (81·0 to 97·1)	26/30	86·7% (69·3 to 96·2)	-4·71% (-21·77 to 8·13)	53/55	96·4% (87·5 to 99·6)	26/26	100·0% (86·8 to 100·0)	3·64% (-9·50 to 12·32)
Fed	407/448	90·8% (87·8 to 93·4)	203/208	97·6% (94·5 to 99·2)	6·75% (2·87 to 10·07)	407/420	96·9% (94·8 to 98·3)	203/203	100·0% (98·2 to 100·0)	3·10% (0·84 to 5·22)
**Baseline resistance status[f]**										
Resistant to azithromycin alone	24/29	82·8% (64·2 to 94·2)	11/12	91·7% (61·5 to 99·8)	8·91% (-19·81 to 27·52)	24/26	92·3% (74·9 to 99·1)	11/11	100·0% (71·5 to 100·0)	7·69% (-18·78 to 24·14)
Resistant to ceftriaxone alone	0	0	0	0	NA	0	0	0	0	NA
Resistant to both ceftriaxone and azithromycin	0	0	0	0	NA	0	0	0	0	NA
Susceptible to both ceftriaxone and azithromycin	435/476	91·4% (88·5 to 93·7)	217/225	96·4% (93·1 to 98·5)	5·06% (1·09 to 8·41)	435/448	97·1% (95·1 to 98·4)	217/217	100·0% (98·3 to 100·0)	2·90% (0·79 to 4·90)
**Received PrEP medication at baseline**										
Yes	65/70	92·9% (84·1 to 97·6)	30/31	96·8% (83·3 to 99·9)	3·92% (-9·67 to 12·83)	65/66	98·5% (91·8 to 100·0)	30/30	100·0% (88·4 to 100·0)	1·52% (-9·90 to 8·10)

	Microbiological cure rate[e]									
	microITT population					Evaluable population				
	Zoliflodacin		Ceftriaxone + azithromycin			Zoliflodacin		Ceftriaxone + azithromycin		
**Subgroup**	**n/N[a]**	**Proportion (95% CI)[b]**	**n/N[a]**	**Proportion (95% CI)[b]**	**Difference (95% CI)[c,d]**	**n/N[a]**	**Proportion (95% CI)[b]**	**n/N[a]**	**Proportion (95% CI)[b]**	**Difference (95% CI)[c,d]**
No	395/436	90·6% (87·5 to 93·2)	199/207	96·1% (92·5 to 98·3)	5·54% (1·24 to 9·18)	395/409	96·6% (94·3 to 98·1)	199/199	100·0% (98·2 to 100·0)	3·42% (1·08 to 5·66)
**Risk factor MSM**										
Yes	153/162	94·4% (89·7 to 97·4)	79/82	96·3% (89·7 to 99·2)	1·90% (-5·16 to 7·15)	153/155	98·7% (95·4 to 99·8)	79/79	100·0% (95·4 to 100·0)	1·29% (-3·44 to 4·58)
No	307/344	89·2% (85·5 to 92·3)	150/156	96·2% (91·8 to 98·6)	6·91% (1·76 to 11·17)	307/320	95·9% (93·2 to 97·8)	150/150	100·0% (97·6 to 100·0)	4·06% (1·06 to 6·83)
**Sex worker**										
Never	432/475	90·9% (88·0 to 93·4)	212/221	95·9% (92·4 to 98·1)	4·98% (0·83 to 8·47)	432/445	97·1% (95·1 to 98·4)	212/212	100·0% (98·3 to 100·0)	2·92% (0·77 to 4·93)
Every now and then (sometimes)	15/18	83·3% (58·6 to 96·4)	15/15	100·0% (78·2 to 100·0)	16·67% (-6·42 to 39·22)	15/17	88·2% (63·6 to 98·5)	15/15	100·0% (78·2 to 100·0)	11·76% (-10·32 to 34·34)
Often	13/13	100·0% (75·3 to 100·0)	2/2	100·0% (15·8 to 100·0)		13/13	100·0% (75·3 to 100·0)	2/2	100·0% (15·8 to 100·0)	
**Age group**										
< 18 years	9/9	100·0% (66·4 to 100·0)	1/1	100·0% (2·5 to 100·0)		9/9	100·0% (66·4 to 100·0)	1/1	100·0% (2·5 to 100·0)	
18 years to 64 years	449/495	90·7% (87·8 to 93·1)	227/236	96·2% (92·9 to 98·2)	5·48% (1·50 to 8·87)	449/464	96·8% (94·7 to 98·2)	227/227	100·0% (98·4 to 100·0)	3·23% (1·14 to 5·26)
≥ 65 years	2/2	100·0% (15·8 to 100·0)	1/1	100·0% (2·5 to 100·0)		2/2	100·0% (15·8 to 100·0)	1/1	100·0% (2·5 to 100·0)	

[a]n: number of participants with cure, N: number of participants in the population for the specified body site and treatment arm. [b]Calculated with the Clopper Pearson method. [c]Calculated with the Newcombe score method. [d]Difference calculated as treatment difference of ceftriaxone + azithromycin minus zoliflodacin. [e]Microbiological cure was defined as a positive *N gonorrhoeae* culture at baseline and negative or indeterminate *N gonorrhoeae* culture at the TOC visit at the urogenital site. [f]Using CLSI criteria.

CI=confidence interval; CLSI=The Clinical and Laboratory Standards Institute; EU=European Union; micro-ITT=microbiological intention-to-treat; MSM=men who have sex with men (i.e., assigned male at birth and usually engage in sexual contact with men or transgender women); NA=not applicable; PrEP=pre-exposure prophylaxis; TOC=test of cure; US=United States.

***Table S10.* Summary of MIC and antimicrobial susceptibility status for *N gonorrhoeae* at TOC: micro-ITT (urogenital) population**

Treatment arm	Antimicrobial	MIC_{50} (µg/mL)	MIC_{90} (µg/mL)	Range (µg/mL)	CLSI[a]		EUCAST[a]	
					MIC susceptible breakpoint	Non-susceptible	MIC susceptible breakpoint	Non-susceptible
Zoliflodacin (M=15)	Azithromycin	0·12	0·25	≤0·06, 2	≤1	6·7%	≤1	6·7%
	Cefixime	0·008	0·015	≤0·002, 0·03	≤0·25	0%	≤0·125	0%
	Ceftriaxone	0·008	0·015	≤0·002, 0·015	≤0·25	0%	≤0·125	0%
	Ciprofloxacin	2	>2	0·004, >2	≤0·06	86·7%	≤0·03	86·7%
	Gentamicin	8	8	4, 8	NA	NA	NA	NA
	Spectinomycin	32	32	16, 32	≤32	0%	≤64	0%
	Tetracycline	>4	>4	0·5, >4	≤0·25	100%	≤0·5	66·7%
	Zoliflodacin	0·06	0·12	≤0·008, 0·25	NA	NA	NA	NA
Ceftriaxone + azithromycin (M=0)	Azithromycin	There were no isolates at TOC in this treatment arm						
	Cefixime							
	Ceftriaxone							
	Ciprofloxacin							
	Gentamicin							
	Spectinomycin							
	Tetracycline							
	Zoliflodacin							

[a]MIC breakpoints obtained from: CLSI criteria[2] and EUCAST criteria.[3]

CLSI=Clinical and Laboratory Standards Institute; EUCAST=European Committee on Antimicrobial Susceptibility Testing; M=number of participants in a micro-ITT population relevant for the specified body site without missing data; MIC=minimum inhibitory concentration; micro-ITT=microbiological intention to treat; MIC_{50}=MIC required to inhibit growth of 50% of isolates; MIC_{90}=MIC required to inhibit growth of 90% of isolates; NA=not applicable; TOC=test of cure.

Table S11. **Summary of MIC and antimicrobial susceptibility status for *N gonorrhoeae* at TOC: micro-ITT (pharyngeal) population**

Treatment arm	Antimicrobial	MIC_{50} (µg/mL)	MIC_{90} (µg/mL)	Range (µg/mL)	CLSI[a]		EUCAST[a]	
					MIC susceptible breakpoint	Non-susceptible	MIC susceptible breakpoint	Non-susceptible
Zoliflodacin (M=4)	Azithromycin	NC	NC	0·12, 4	≤1	75%	≤1	75%
	Cefixime	NC	NC	0·008, 0·015	≤0·25	0%	≤0·125	0%
	Ceftriaxone	NC	NC	0·008, 0·015	≤0·25	0%	≤0·125	0%
	Ciprofloxacin	NC	NC	0·004, >2	≤0·06	25·0%	≤0·03	25·0%
	Gentamicin	NC	NC	4, 8	NA	NA	NA	NA
	Spectinomycin	NC	NC	32, 32	≤32	0%	≤64	0%
	Tetracycline	NC	NC	0·25, 4	≤0·25	75·0%	≤0·5	75·0%
	Zoliflodacin	NC	NC	0·12, 0·25	NA	NA	NA	NA
Ceftriaxone + azithromycin (M=1)	Azithromycin	NC	NC	0·12, 0·12	≤1	0%	≤1	0%
	Cefixime	NC	NC	0·06, 0·06	≤0·25	0%	≤0·125	0%
	Ceftriaxone	NC	NC	0·008, 0·008	≤0·25	0%	≤0·125	0%
	Ciprofloxacin	NC	NC	>2, >2	≤0·06	100%	≤0·03	100%
	Gentamicin	NC	NC	4, 4	NA	NA	NA	NA
	Spectinomycin	NC	NC	32, 32	≤32	0%	≤64	0%
	Tetracycline	NC	NC	>4, >4	≤0·25	100%	≤0·5	100%
	Zoliflodacin	NC	NC	0·06, 0·06	NA	NA	NA	NA

[a]MIC breakpoints obtained from: CLSI criteria[2] and EUCAST.[3]

CLSI=Clinical and Laboratory Standards Institute; EUCAST=European Committee on Antimicrobial Susceptibility Testing; M=number of participants in a micro-ITT population relevant for the specified body site without missing data; MIC=minimum inhibitory concentration; micro-ITT=microbiological intention to treat; MIC_{50}=MIC required to inhibit growth of 50% of isolates; MIC_{90}=MIC required to inhibit growth of 90% of isolates; NA=not applicable; NC=not calculated; TOC=test of cure.

Table S12. **Summary of MIC and antimicrobial susceptibility status for *N gonorrhoeae* at TOC: micro-ITT (rectal) population**

Treatment arm	Antimicrobial	MIC_{50} (µg/mL)	MIC_{90} (µg/mL)	Range (µg/mL)	CLSI[a]		EUCAST[a]	
					MIC susceptible breakpoint	Non-susceptible	MIC susceptible breakpoint	Non-susceptible
Zoliflodacin (M=3)	Azithromycin	NC	NC	≤0·06, 0·12	≤1	0%	≤1	0%
	Cefixime	NC	NC	0·004, 0·015	≤0·25	0%	≤0·125	0%
	Ceftriaxone	NC	NC	0·004, 0·004	≤0·25	0%	≤0·125	0%
	Ciprofloxacin	NC	NC	1, 2	≤0·06	100%	≤0·03	100%
	Gentamicin	NC	NC	8, 16	NA	NA	NA	NA
	Spectinomycin	NC	NC	16, 32	≤32	0%	≤64	0%
	Tetracycline	NC	NC	>4, >4	≤0·25	100%	≤0·5	100%
	Zoliflodacin	NC	NC	≤0·008, ≤0·008	NA	NA	NA	NA
Ceftriaxone + azithromycin (M=0)	Azithromycin	There were no isolates at TOC in this treatment arm						
	Cefixime							
	Ceftriaxone							
	Ciprofloxacin							
	Gentamicin							
	Spectinomycin							
	Tetracycline							
	Zoliflodacin							

[a]MIC breakpoints obtained from: CLSI criteria[2] and EUCAST criteria.[3]

CLSI=Clinical and Laboratory Standards Institute; EUCAST=European Committee on Antimicrobial Susceptibility Testing; M=number of participants in a micro-ITT population relevant for the specified body site without missing data; MIC=minimum inhibitory concentration; micro-ITT=microbiological intention to treat; MIC_{50}=MIC required to inhibit growth of 50% of isolates; MIC_{90}=MIC required to inhibit growth of 90% of isolates; NA=not applicable; TOC=test of cure.

Table S13. N gonorrhoeae NAAT results at TOC (all populations and anatomical sites)

Anatomical site	Analysis population	NAAT result category[a]	Zoliflodacin n/N (%)	Ceftriaxone + azithromycin n/N (%)
**Urogenital**	Micro-ITT[b]	Total	492	233
		Positive	65/492 (13·2)	30/233 (12·9)
		Negative	405/492 (82·3)	190/233 (81·5)
		Indeterminate	5/492 (1·0)	6/233 (2·6)
		Not completed[c]	2/492 (0·4)	1/233 (0·4)
	Evaluable	Total	463	225
		Positive	64/463 (13·8)	30/225 (13·3)
		Negative	394/463 (85·1)	189/225 (84·0)
		Indeterminate	5/463 (1·1)	6/225 (2·7)
		Not completed[c]	0/463	0/225
	Per-protocol	Total	440	215
		Positive	56/440 (12·7)	28/215 (13·0)
		Negative	378/440 (85·9)	180/215 (83·7)
		Indeterminate	5/440 (1·1)	6/215 (2·8)
		Not completed[c]	1/440 (0·2)	1/215 (0·5)
**Pharyngeal**	Micro-ITT[b]	Total	45	24
		Positive	11/45 (24·4)	8/24 (33·3)
		Negative	28/45 (62·2)	12/24 (50·0)
		Indeterminate	3/45 (6·7)	2/24 (8·3)
		Not completed[c]	2/45 (4·4)	1/24 (4·2)
	Evaluable	Total	39	21
		Positive	11/39 (28·2)	7/21 (33·3)
		Negative	26/39 (66·7)	12/21 (57·1)
		Indeterminate	2/39 (5·1)	2/21 (9·5)
		Not completed[c]	0/39	0/21
	Per-protocol	Total	39	20
		Positive	10/39 (25·6)	7/20 (35·0)
		Negative	25/39 (64·1)	10/20 (50·0)
		Indeterminate	3/39 (7·7)	2/20 (10·0)
		Not completed[c]	1/39 (2·6)	1/20 (5·0)
**Rectal**	Micro-ITT[b]	Total	73	30
		Positive	8/73 (11·0)	1/30 (3·3)
		Negative	60/73 (82·2)	27/30 (90·0)
		Indeterminate	1/73 (1·4)	1/30 (3·3)
		Not completed[c]	1/73 (1·4)	0/30
	Evaluable	Total	66	28
		Positive	7/66 (10·6)	1/28 (3·6)
		Negative	58/66 (87·9)	26/28 (92·9)
		Indeterminate	1/66 (1·5)	1/28 (3·6)
		Not completed[c]	0/66	0/28
	Per-protocol	Total	58	27
		Positive	6/58 (10·3)	1/27 (3·7)
		Negative	51/58 (87·9)	25/27 (92·6)
		Indeterminate	1/58 (1·7)	1/27 (3·7)
		Not completed[c]	0/58	0/27

[a]NAAT results were automatically determined by the assay software. An indeterminate result was only declared after a repeat analysis. [b]Data for the Modified Micro-ITT population were identical to the Micro-ITT population and are not shown. [c]For all isolates the reason for "not completed" was "not done".

Micro-ITT=microbiological intention-to-treat; NAAT=nucleic acid amplification test; n=number of participants in category; TOC=test of cure.

Table S14. **Summary of absolute neutrophil count by visit and race: safety population**

Race[a]	Visit	Stat	Zoliflodacin		CTR-AZI		Overall	
			Value	Change	Value	Change	Value	Change
			Absolute neutrophil count ´ 10^9/L					
Black or African American	Baseline	N	348	-	164	-	512	-
		Mean (SD)	3.8 (1.9)	-	3.7 (1.8)	-	3.8 (1.8)	-
		Median (Min-max)	3.3 (1.1 – 16.6)	-	3.4 (1.1 – 13.6)	-	3.4 (1.1 – 16.6)	-
	EOT	N	310	310	153	153	463	463
		Mean (SD)	2.8 (1.6)	-1.1 (1.6)	2.6 (1.6)	-1.1 (1.8)	2.7 (1.6)	-1.1 (1.7)
		Median (Min-max)	2.4 (0.4 – 9.7)	-0.8 (-8.2 – 5.0)	2.2 (0.7 – 8.8)	-1.0 (-10.1 – 4.7)	2.3 (0.4 – 9.7)	-0.9 (-10.1 – 5.0)
Asian	Baseline	N	193	-	92	-	285	-
		Mean (SD)	5.8 (2.1)	-	5.9 (2.0)	-	5.8 (2.1)	-
		Median (Min-max)	5.5 (1.9 – 14.6)	-	5.2 (2.4 – 11.9)	-	5.4 (1.9 – 14.6)	-
	EOT	N	187	187	88	88	275	275
		Mean (SD)	3.7 (1.3)	-2.1 (2.1)	3.6 (1.2)	-2.3 (2.0)	3.7 (1.3)	-2.1 (2.1)
		Median (Min-max)	3.5 (1.4 – 10.2)	-1.9 (-9.5 – 2.9)	3.6 (1.4 - 6.6)	-1.9 (-8.1 – 1.0)	3.6 (1.4 – 10.2)	-1.9 (-9.5 – 2.9)
White	Baseline	N	65	-	47	-	112	-
		Mean (SD)	5.1 (1.7)	-	5.1 (1.8)	-	5.1 (1.7)	-
		Median (Min-max)	4.8 (2.7 – 10.6)	-	4.9 (1.8 – 9.5)	-	4.8 (1.8 – 10.6)	-
	EOT	N	54	54	37	37	91	91
		Mean (SD)	3.3 (1.2)	-1.6 (1.5)	3.9 (1.6)	-1.3 (2.4)	3.6 (1.4)	-1.5 (1.9)
		Median (Min-max)	3.2 (1.4 – 7.8)	-1.4 (-5.4 – 1.2)	3.4 (1.5 – 7.4)	-0.9 (-6.5 – 3.6)	3.3 (1.4 – 7.8)	-1.4 (-6.5 – 3.6)

[a]Only Black of African American, Asian and White races shown. Other categories had too few participants for meaningful assessment

CTR-AZI, ceftriaxone-azithromycin; EOT, end of trial; min-max, minimum-maximum; N, number of participants in each race category at specified study visit; SD, standard deviation

References

1. FDA. Uncomplicated Gonorrhea: Developing Drugs for Treatment - Guidance for Industry. 2015. https://fda.report/media/88904/Uncomplicated-Gonorrhea--Developing-Drugs-for-Treatment.pdf (accessed 23 May 2025).
2. CLSI. M100 - Performance Standards for Antimicrobial Susceptibility Testing, 32nd Edition. 2022. https://clsi.org/shop/standards/m100/ (accessed 23 May 2025).
3. EUCAST. Breakpoint tables for interpretation of MICs and zone diameters Version 12.0. 2022. https://www.eucast.org/fileadmin/src/media/PDFs/EUCAST_files/Breakpoint_tables/v_12.0_Breakpoint_Tables.pdf (accessed 23 May 2025).
